# Divide and conquer approach for genome-wide association studies

**DOI:** 10.1093/genetics/iyaf019

**Published:** 2025-03-13

**Authors:** Mustafa İsmail Özkaraca, Mulya Agung, Pau Navarro, Albert Tenesa

**Affiliations:** The Roslin Institute, The University of Edinburgh, Edinburgh EH25 9RG, UK; The Institute of Genetics and Cancer, The University of Edinburgh, Edinburgh EH4 2XU, UK; The Institute of Genetics and Cancer, The University of Edinburgh, Edinburgh EH4 2XU, UK; The Roslin Institute, The University of Edinburgh, Edinburgh EH25 9RG, UK; The Roslin Institute, The University of Edinburgh, Edinburgh EH25 9RG, UK; The Institute of Genetics and Cancer, The University of Edinburgh, Edinburgh EH4 2XU, UK

**Keywords:** Genome-wide association studies (GWAS), meta-analysis, population structure, winner's curse

## Abstract

Genome-wide association studies (GWAS) are computationally intensive, requiring significant time and resources with computational complexity scaling at least linearly with sample size. Here, we present an accurate and resource-efficient pipeline for GWAS that mitigates the impact of sample size on computational demands. Our approach involves (1) randomly partitioning the cohort into equally sized sub-cohorts, (2) conducting independent GWAS within each sub-cohort, and (3) integrating the results using a novel meta-analysis technique that accounts for population structure and other confounders between sub-cohorts. Importantly, we demonstrate through simulations and real-data examples in humans that our approach effectively manages analyzing related individuals, a critical factor in real datasets, while controlling for inflated effect sizes, a phenomenon known as winner's curse. We show that our method achieves the same discovery levels as standard approaches but with significantly reduced computational costs. Additionally, it is well-suited for incremental GWAS as new samples are added over time. Our implementation within a bioinformatics workflow management system enhances reproducibility and scalability.

## Introduction

The fundamental goal of genome-wide association studies (GWAS) is to detect associations between genotypes and phenotypes to elucidate the underlying genetic architecture of traits. Many thousands of associations between genotypes and complex traits have been successfully reported (MacArthur *et al*. 2017; Abdellaoui *et al*. 2023) since the first GWAS in 2005 (Klein *et al*. 2005). However, because single genotype variants explain little variance for complex polygenic traits (Yang *et al*. 2010; Boyle *et al.* 2017), studies require large samples to achieve sufficient statistical power to pass desired significance thresholds (Gibson 2010; Ball 2013).

With the advent of cheap(er) genotyping and sequencing techniques, large(r) human genotype datasets have become available for researchers, lifting the curse of low statistical power to detect loci linked to complex traits. On the other hand, analyzing these mega-large datasets comes at a price. Large cohort studies require vast computational resources; processing time and memory space. On top of that, there is a concern of the security and maintenance of sensitive personal information of large-scale cohorts, which ties the hands of researchers to use specific online platforms such as (DNAnexus; UKBiobank-RAP), or secure shared computing clusters such as (ECDF) or else like (ARCHER2), whenever necessary permissions are secured from associated biobanks. All these environments have particular built-in computing infrastructures and require a substantial budget to be able to analyze large-scale datasets, which is only accessible to some researchers with generous funding. Here, we study how to ease the problems associated with ever-increasing sample sizes from a computational and statistical perspective. This will reduce the cost of large-scale GWAS and help make it available to a wider research community. To increase the scalability and reproducibility of our approach, we implemented it in a bioinformatics workflow system extending analysis routines from 2 exemplar GWAS methods: REGENIE (Mbatchou *et al*. 2021) and fastGWA-GLMM (Jiang *et al*. 2021).

Our proposed tool uses a “Divide” and “Conquer” approach. Precisely, we split a given dataset into smaller cohorts equally and randomly, conduct GWAS in each cohort independently through the user's method of choice (i.e. either REGENIE or fastGWA-GLMM), and finally combine summary results from each sub-cohort using our new method, which we call *corrected-meta-analysis* (CMA). Since GWAS in each sub-cohort can be implemented in parallel, this pipeline alleviates the effect of sample size on complexity equations, allowing users to run GWAS even with less computational resources, and yet gain substantial time. Furthermore, we have shown through both simulations and real data examples that our pipeline, when compared with standard GWAS methodology such as (Jiang *et al*. 2021; Mbatchou *et al*. 2021), is adequate for controlling inflation of effect sizes and providing the same level of true discovery while faster and less resource-demanding than existing approaches.

## Results

Our approach decouples the predictivity (i.e. estimation of effect sizes) and discovery (i.e. detecting loci) parts of GWAS analyses. We conducted comprehensive comparisons between 3 approaches: standard GWAS (***Standard***), standard fixed-effect meta-analysis (***MA***), and our proposed approach, referred to as corrected meta-analysis (***CMA***), under different predictivity and discovery scenarios. We simulated 560 continuous traits using GCTA (version 1.94.0 beta Linux) (Yang *et al*. 2011) under 28 different cases, each of which consists of 20 phenotypes (Supplementary Table 1). Additionally, 12 real traits from the UK Biobank (UKB) (Bycroft *et al*. 2018)—7 continuous and 5 diseases—have been analyzed (Supplementary Table 2). All simulated and real traits have been used to examine the performances of the outlined approaches under various settings.

### Predictivity

A total of 560 simulated traits have been used to assess bias in estimating effects. GWAS is conducted for each of the 560 traits using the Standard, MA, and CMA methods. For each GWAS, MA, and CMA have been tested by dividing the entire cohort into *k* equal cohorts randomly, with *k* taking the values of 2, 4, 8, 16, 32, and 64. This leads to 6 GWAS solutions (one for each value of *k*) for each trait for both the MA and the CMA methods. Consequently, a total of 13 different scenarios were assessed, including the standard GWAS approach (Standard). The accuracy of effect sizes was then investigated under 3 distinct metrics:

1.
$R^{2}$ *between* $\beta_{true}$ *and* $\hat{\beta }$ : This approach is equivalent of comparing mean squared errors between true and predicted values between different methods.
2. *Quantification of winner's curse:* We call the fraction $\gamma =\;\hat{\beta }/\beta_{\mathrm{true}}$*effect inflation factor* (EIF). It is >1 whenever the associated variant effect is upwardly biased (i.e. winner's curse for that variant). This fraction is measured for each causal variant and then integrated into a single metric $\mu_{\gamma }=(\sum |1-\gamma_{i}|)/n,$ where *n* is the number of causal variants. We use $\mu_{\gamma }$ to assess the winner's curse between different methods.
3. *Polygenic Risk Score (PRS) estimates:* PRS analyses have been implemented on a target cohort (20% of the data), using GWAS summary results from the discovery cohort (80% of the data).

**Fig. 1.**
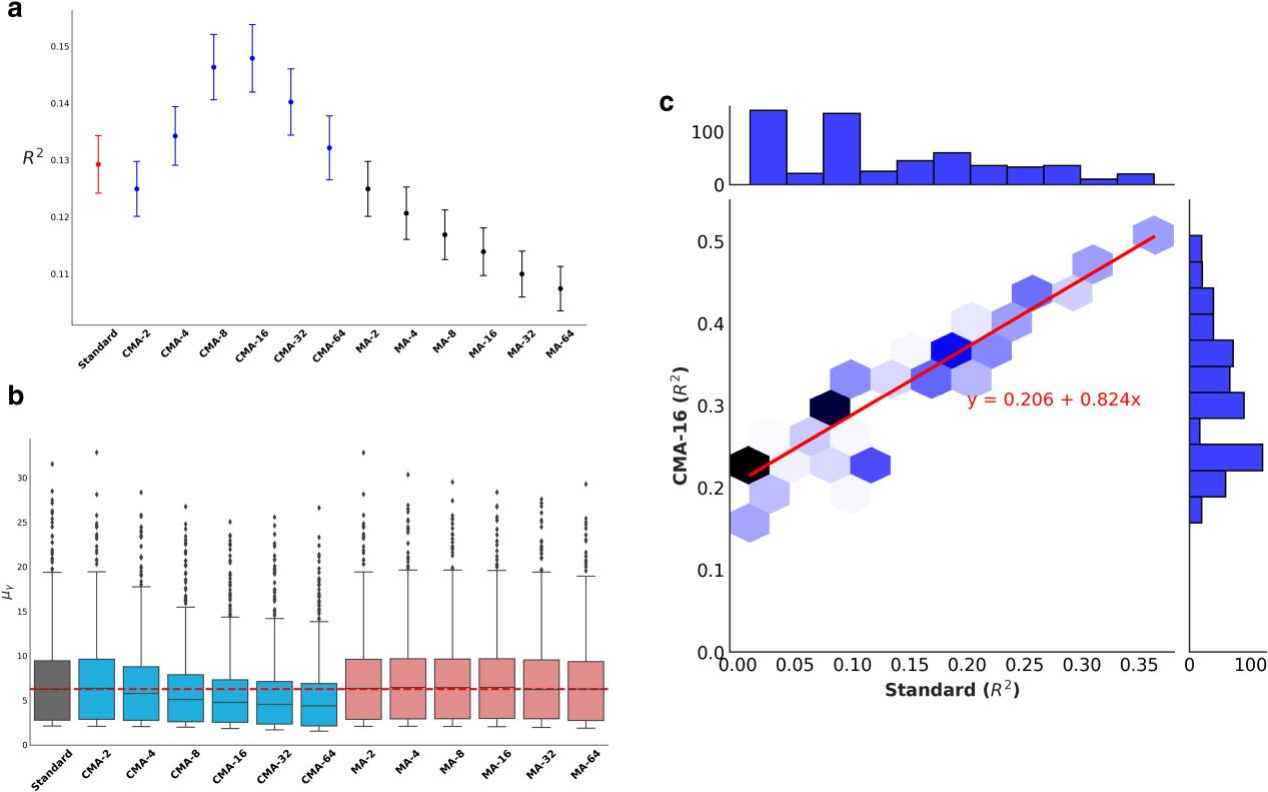
Thirteen different analyses are assessed. CMA-x (or MA-x) refers to the corrected meta-analysis (or meta-analysis) method, which divides entire cohort into × equal cohorts randomly. a) Effect size estimates are assessed under the model $\beta_{\mathrm{true}}∼\hat{\beta }+\epsilon$. The coefficient of determination ($R^{2}$) value of the models is computed. Distributions of $R^{2}$ values are presented through the intervals [μ−1.96*σ/N,μ+1.96*σ/N], where *μ* is the mean of $R^{2}$ values, *σ* is the standard deviation of $R^{2}$ values and *N* is the number of causal variants in a simulation. CMA based methods outperformed other methods for each case with split index x≥4. b) Distribution of inflation of effect size estimates demonstrated through the metric $\mu_{\gamma }$. CMA-based methods outperform the others we assessed by yielding lesser inflation on effect size estimates, indicating that our approach is controlling winner's curse better than the alternatives. The horizontal line denotes the median of $\mu_{\gamma }$ values for S (Standard). c) PRS estimations have been assessed through the model y∼PRS+ϵ. The coefficients of determination ($R^{2}$) of the models are compared for evaluation. CMA-based approaches outperformed the other methods. The graph only shows the comparison of CMA-16 with S, demonstrating that there is a high correlation between $R^{2}$ values of S and CMA-16, with the vital difference that CMA-16 on average explains ∼20% more variation than S for lower heritable traits and about 15% more variation than S for higher heritable traits.

For all 3 metrics, our method (with any choice of split index k≥4) outperformed other methods significantly, which validates that our approach has better effect size estimators compared with standard GWAS and highlights the fact that standard meta-analysis approaches suffer from loss of information between cohorts and should be avoided when there is a possibility that there are related individuals between cohorts (e.g. as we approach population-wide genetic testing). These results are shown in Fig. 1.

Meta-analysis-based methods are suitable for updating summary results as data are generated and added to the database to be analyzed. We further tested the predictivity performance of our approach under this setting by focusing on 20 simulated phenotypes with a heritability of 0.3 and 5,000 causal variants randomly chosen from genotype data (Supplementary Table 1—Cell B5). We tested 6 different meta-analysis scenarios (Supplementary Table 2). For example, scenario (*d*) (320+80) in Supplementary Table 2 indicates that 320,000 individuals have already been analyzed, and 80,000 more individuals are being added later. We split the latter cohort into 8 sub-cohorts, each of which contains 10,000 individuals, and run GWAS in each cohort independently. Lastly, we combine 9 (8 + 1) sets of summary results using both meta-analysis techniques, ***CMA*** and ***MA***, respectively. Supplementary Fig. 1 shows comparisons of effect size estimates obtained from those scenarios. The figure shows that our method yields accurate incremental GWAS results while standard MA approaches suffer from loss of accuracy.

### Discovery

**Fig. 2.**
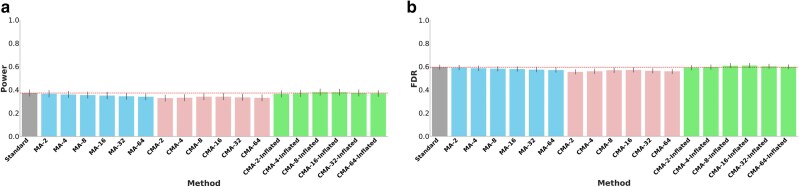
Nineteen different methods are assessed for power a) and FDR b) over 560 simulated traits. CMA methods with power-inflation feature are shown as CMA-X-Inflated, where X is the number of sub-cohorts. CMA methods with power-inflation feature reach the same level of both power and FDR as the Standard approach.

**Fig. 3.**
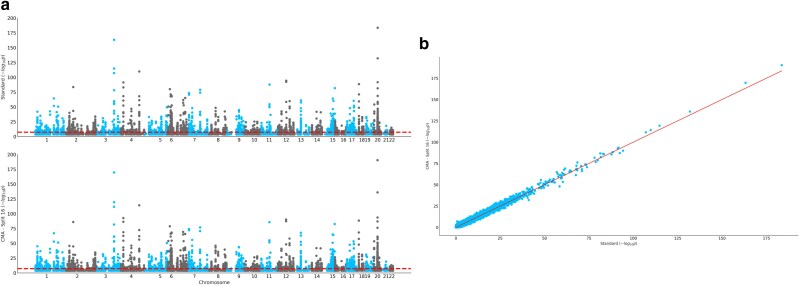
a) Manhattan plots for height are shown for Standard and CMA-16. Both methods discover the same loci as significantly associated with corresponding traits. b) QQ plot for log_10_*P*-values obtained from both methods for height. Both methods have same level of discovery.

We calculated the statistical power and false discovery rates (FDR) of each method for each GWAS implemented on 560 simulated traits. CMA has been assessed with and without our power inflation feature (**Methods**). Figure 2 outlines power and FDR comparisons. CMA with the power-inflation feature reached the same level of both power and FDR rates as Standard with genomic control applied. Additionally, we conducted GWAS on 12 real traits from UKB; 7 continuous and 5 disease traits (Supplementary Table 3). We tested our method for discovery using the power inflation feature (**Methods**) with split index 16, using SNP-heritability predictions from Canela-Xandri *et al.* (2018) (Supplementary Table 3). CMA and the standard approach (with genomic control) are equivalent as they detect the same loci for each trait. These results can be seen from Manhattan and QQ plots comparing the CMA-16 and Standard (Fig. 3 and Supplementary Figs. 2–12).

### Statistical performance of related individuals

To quantify the statistical performance of CMA on related individuals in comparison with other tools, including REGENIE and fastGWA-GLMM, we simulated quantitative and binary traits using the real UKB genotype data. We sampled individuals from the white British ancestry set of the UKB and selected one of the following: (1) the whole set containing a mix of unrelated and related individuals (*N* = 409,204); (2) only unrelated individuals (*N* = 342,587); (3) only related individuals (*N* = 66,617). To consider samples with higher amounts of relatedness, we sampled first- to third-degree relatives in the set of white British participants. Using the sampled individuals, we simulated quantitative and binary traits using the GCTA software. For different methods, we quantify the false positive ratio (FPR) using the proportion of null variants with *P*-values less than a threshold. To quantify power, we used the proportion of causal variants with *P*-values less than a threshold, that is, the proportion of causal variants identified. More details can be found in the section “Data simulation for statistical performance on related individuals.”

We implemented CMA as 2 Snakemake workflows that extend 2 existing GWAS tools, REGENIE (R + CMA) for quantitative and binary traits and fastGWA-GLMM (F + CMA) for binary traits. We use the GWAS tools to perform association testing for each sub-cohort. The workflows are implemented to take advantage of the sequential and parallel computations of CMA. We detail our implementation in **Materials and Methods**.

For quantitative traits, we compared R + CMA with REGENIE across simulation replicates and different numbers of causal variants. With similar empirical power results, R + CMA had slightly lower false-positive rates (Supplementary Fig. 13). The largest differences in false-positive rates occur in the scenario with related individuals. The lower FPR was also observed in the mix of unrelated-related individuals, indicating that CMA can better calibrate the test statistics with related individuals. For binary traits, we compared R + CMA and F + CMA with REGENIE and fastGWA-GLMM across simulation replicates and different case-control ratios. R + CMA and REGENIE had similar false-positive rates and power across the relatedness scenarios (Supplementary Fig. 14). This was also observed when comparing F + CMA and fastGWA-GLMM, showing that CMA maintained FPR and power even after splitting the cohort. In addition, F + CMA had the lowest FPR among the methods for the scenarios with mixed unrelated-related individuals and unrelated individuals, showing that CMA calibrates the test statistics better also with unrelated individuals.

### Computational performance results

To demonstrate the computational advantages of CMA, we compared the runtimes and memory usages of R + CMA and F + CMA to REGENIE and fastGWA-GLMM, respectively. For the R + CMA comparison, we used simulated quantitative and binary traits with the UKB genotype array and simulated genotypes for 5 million individuals. For the F + CMA comparison, we used simulated a binary traits and genotype dataset of 2 million individuals. We used a smaller sample size for the F + CMA comparison due to the computational time constraints of fastGWA-GLMM. Generating the GRM matrix for 2 million individuals took ∼2,056 processing unit (CPU) hours.

**Fig. 4.**
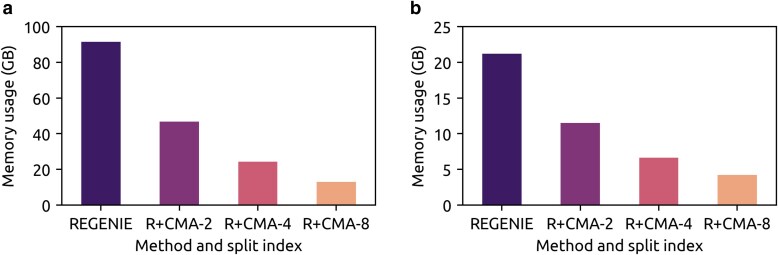
The memory usages of R + CMA and REGENIE for steps 1 and 2 split indexes 2, 4, and 6 for analyzing 10 quantitative and 10 binary simulated traits with the UKB data. a) step 1 was run with block size = 1,000, and (b) step 2 was run with block size = 2,000. Both steps were run using the serial strategy on a 16-core Intel Xeon 2.4 GHz CPU with 96 GB RAM.

We compared the computational performances of R + CMA and REGENIE on a set of 10 quantitative traits and 10 binary traits from the UKB data with ∼400,000 individuals. We set the block size parameters for steps 1 and 2 as 1,000 and 2,000, respectively. Figure 4 shows the memory usages of R + CMA and REGENIE. For R + CMA, the memory usage was measured for each sub-cohort with the serial strategy (Supplementary Fig. 20a). As shown in the figure, CMA reduced the memory usage proportionally to the split index or the number of sub-cohorts. For instance, the R + CMA-4 reduced the memory usage of REGENIE by 4-fold. The computational runtimes and compute costs of the 2 methods are given in Table 1. The runtime was measured when computing different sub-cohorts in parallel on a single node. As shown in the table, the proposed method was up to 3.06-fold faster than REGENIE. The speed-up ratios for analyzing quantitative and binary traits are shown in Supplementary Fig. 15. R + CMA achieved a higher speed-up in step 1, indicating that step 1 computation is more CPU intensive than step 2 computation. Thus, R + CMA can gain a higher CPU utilization in step 1 (Supplementary Fig. 16). We estimated the compute cost on the UKB Research Analysis Platform (UKB RAP) by calculating the price rates on a single instance of the mem2_ssd1_v2_x16 type. As shown in the table, R + CMA reduces the compute cost accordingly with the speed-up.

**Table 1.** Computational performance and compute costs of R + CMA and REGENIE for analyzing 10 simulated quantitative traits and 10 simulated binary traits with UKB data.

Method	REGENIE step	Quantitative trait (QT) analysis runtime (h)	Binary trait (BT) analysis runtime (h)	RAP compute cost of running 100× analyses (QT/BT)
REGENIE	1	2.3	7.3	£110/£360
	2	0.5	0.8	£30/£390
R + CMA-2	1	1.2	4.3	£60/£210
	2	0.4	0.6	£20/£30
R + CMA-4	1	0.9	3.1	£42/£150
	2	0.3	0.4	£11/£24
R + CMA-8	1	0.7	2.6	£37/£13
	2	0.2	0.3	£9.8/£19

For both methods, 505,977 LD-pruned SNPs were used for fitting the null model (step 1). For binary traits, step 2 was run with Firth correction. All the runs were performed on 16 CPU cores of a 2.4 GHz Intel E5-2630 v3 processor. The compute cost is the price rate calculated from the 100 replicated runtimes and the hourly rate of mem2_ssd1_v2_x16 instance type on the UKB RAP.

As step 1 needs larger memory space than step 2, one could use a small subset of variants or genotype array in step 1 (Mbatchou *et al*. 2021). However, even with a small number of variants, performing step 1 is still computationally challenging with a large sample size. We used the simulated dataset of 5 million individuals (**Materials and Methods**) to show the advantage of CMA in conducting GWAS with large sample sizes. We compared the memory usage of R + CMA and REGENIE by running step 1 with a predefined subset of ∼80,000 variants not in linkage disequilibrium and step 2 with all the variants. The block size parameters are set the same as in the computational performance evaluation with the UKB dataset.

As shown in Supplementary Fig. 17, REGENIE still requires a larger memory space in step 1 than in step 2 despite the smaller number of variants because step 1 needs to load more than one marker block to memory to fit a whole-genome regression model. Consistent with the results of the UKB data, R + CMA reduces the memory usage linearly with split index. As shown in the R + CMA-16 results, splitting the cohort into 16 sub-cohorts reduced the memory usage from 200 to 13 GB, which means that R + CMA-16 can use a computing node equipped with a 16-fold smaller memory capacity than the required memory capacity for REGENIE. The runtime of running R + CMA-16 in parallel was up to 9.4-fold and 5.2-fold faster than REGENIE for steps 1 and 2, respectively (Supplementary Fig. 18).

For F + CMA, we compared the memory usage and runtime results of the GLMM fitting step (Supplementary Fig. 19) with the simulated dataset of 2 million individuals (**Materials and Methods**). F + CMA reduced the memory usage almost linearly with the split index. For runtime, F + CMA can achieve a speed-up of 3.5-fold compared with fastGWA-GLMM. The runtime results show that even in a single node system, both the CMA implementations can achieve a notable speed-up by dividing the cohort and computing different sub-cohorts in parallel. R + CMA and F + CMA reduced the compute costs on ARCHER2 and the UKB RAP accordingly with the speed-up (Supplementary Table 4).

## Discussion

Over the last few decades, genotyping and sequencing costs have substantially decreased, which has led to hundreds of thousands of individuals being genotyped to discover trait-associated loci through GWAS. Many resource-efficient algorithms have been developed to accommodate large-scale datasets in GWAS analyses (Canela-Xandri *et al*. 2015; Loh *et al*. 2015; Zhou *et al*. 2018; Jiang *et al*. 2019, 2021; Mbatchou *et al*. 2021). GWAS software REGENIE (Mbatchou *et al*. 2021) uses a machine learning approach based on dividing variants into mutually disjoint blocks. This approach motivated us to investigate partitioning cohorts and applying meta-analysis to combine separate GWAS summary statistics obtained from sub-cohorts.

Splitting a cohort into multiple sub-cohorts and applying standard meta-analysis techniques (i.e. inverse variance method) interprets analyses in sub-cohorts as mutually independent. This is a strong assumption and ignores population structure as well as other possible confounding factors between cohorts. It is violated whenever there are related individuals separated between different cohorts, a typical scenario for GWAS. As such, the inverse-variance-based meta-analysis approach, although computationally efficient due to its nature of splitting the analysis into smaller parts, is not preferred over standard GWAS. In general, GWAS of a full cohort is the gold standard for the task of detecting causal variants associated to complex traits (Stewart and Tierney 2002). Here, we propose a novel meta-analysis method (corrected meta-analysis) that assumes GWAS analyses between cohorts are (mutually) dependent. By estimating and correcting for this cohort dependency information between cohorts, we are able to improve the accuracy and predictivity of summary results.

Our “divide” and “conquer” approach is implemented on 2 levels: within cohorts and between cohorts. The former is done with current gold standard open-source GWAS software such as REGENIE and fastGWA-GLMM, depending on the user's choice. Any confounding factor in a (sub-)cohort GWAS is corrected by REGENIE/fastGWA-GLMM for “within cohort” analyses. On the other hand, any confounding factor “between cohorts,” as well as loss of genetic information “between cohorts” is corrected by corrected meta-analysis. The former step is based on classic assumptions, such as that the true underlying model is an additive genetic model, whereas the latter step is independent of those assumptions and is valid even if the true underlying model is different than what has been assumed. We have demonstrated in this paper that our method corrects for population structure and other possible confounding factors even better than standard GWAS analysis. As such, the use of already available summary statistics through our meta-analysis approach becomes more beneficial for prospective GWAS, minimizing the need to share genotype information of large-scale cohorts. Additionally, by decoupling the predictivity and discovery aims of GWAS, we are also able to detect the same loci as with the standard approach (Fig. 3 and Supplementary Figs. 2–12). That is, we are not penalizing power or compromising on the desired FDR levels when using our approach, unlike when implementing standard inverse-variance meta-analysis with hidden structure between cohorts.

Lastly, our proposed method is built as an efficient bioinformatics workflow and is distributed as open-source. It is easy to use on multiple computing platforms. Our software also facilitates memory and resource efficiency at its best due to the partitioning cohort argument, offering a powerful alternative to already available standard GWAS approaches. For example, our method could be 9.4-fold and 5.2-fold faster than REGENIE—the fastest large-scale GWAS software for continuous traits to date—for steps 1 and 2, respectively (Supplementary Fig. 18). In addition, our method can use a computing instance equipped with ∼16-fold smaller memory space than what would be required for implementing GWAS with REGENIE and cost significantly less (Supplementary Fig. 17 and Supplementary Table 4). This feature is especially vital in cloud computing networks where a single (or multiple) GWAS analysis (analyses) can be completed using cheaper instances of users’ choice, rather than requesting/waiting for large instances to be allocated or the analyses being interrupted due to insufficient resources.

As sample size increases, it becomes vital to address the resulting impact on computational efficiency. CMA is currently the only resource economic method capable of addressing this issue. Given the significant improvements our method offers in efficiency and scalability, we anticipate that further advances in developing efficient software in GWAS will adopt our approach to mitigate the challenges associated with large cohort sizes.

## Materials and methods

CMA is essentially an extension of the work of Lin and Sullivan (2009), which focuses on handling overlapping samples between cohorts. The key innovation of CMA is its ability to explicitly incorporate the relatedness structure of overlapping samples across subgroups rather than merely correcting for overlap. This approach enhances between-study adjustments for potential confounding effects between sub-cohorts. For example, for cohort 1 = [ID1, ID2, ID3] and cohort 2 = [ID4, ID5, ID6], imagine 3 different scenarios of meta-analysis: (A) cohort 1 and cohort 2 consist of unrelated individuals with one overlapping sample [ID1], (B) cohort 1 and cohort 2 consist of unrelated individuals with no overlapping samples, and (C) cohort 1 and cohort 2 consist of nonoverlapping unrelated individuals except that ID1 (from cohort 1) and ID6 (from cohort 2) are siblings.

Lin and Sullivan (2009) showed that the same (inverse-variance) method for scenario A and scenario B should not be used. Indeed, the inverse-variance method would cause inflation of type I error rate had it been used for scenario A, due to ID1 being double counted. Similarly, scenario C is a case where cohorts have no overlapping samples, but there is a pair of siblings separated between cohorts. Approximately half of the input information obtained from those siblings is the same. Therefore, the same logical inference Lin and Sullivan (2009) suggest for scenario A should be extended to scenario C to control the inflation of type I error rate. We refer to this phenomenon as *overlapping relationships* between cohorts. As such, we extend the approach in Lin and Sullivan (2009) to account for this issue by allowing the overlapping sample variable $\hat{n_{12}}$ in Equation 8 in Lin and Sullivan (2009) to be any real number, rather than only an integer number quantifying the number of overlapping samples between cohorts.

We explain our method with a simple case of splitting a cohort into 2. Let $\beta_{i},SE_{i},z_{i},Coh_{i}$ denote effect size estimate, its standard error, *z*-score and collection of all individuals in the cohort *i*, for i=1,2. Using the same notations and formulas in Lin and Sullivan (2009), we are interested in estimating $Corr(\beta_{1},\beta_{2}),$ where $\beta_{1},\beta_{2}$ are marker effect estimates obtained from different studies. Precisely, we are interested in finding a variable $\hat{n_{12}}$ that accounts for relationship overlap so that $Corr(\beta_{1},\beta_{2})=\hat{n_{12}}/(\sqrt{n_{1}n_{2}}),$ where $n_{1},n_{2}$ denote sample sizes.

We use a similar argument explained in (METAL) by the authors of Willer *et al.* (2010). There are 2 cases: (i) Variant-*i* is a null SNP and (ii) Variant-*i* is not a null SNP.

Suppose first that $\beta_{i},SE_{i}$ for i=1,2 are obtained for a null SNP in the genotype data. We have $z_{1},z_{2}∼N(0,1)$. Assume further that there are related individuals separated between sub-cohorts. We partition each sub-cohort into 2 groups; unrelated and related individuals. Related individuals correspond to pairs of individuals with high kinship coefficients and separated between distinct cohorts. For example, for scenario C above, related individuals for cohort 1 are/is the collection [ID1], whereas related individuals for cohort 2 are/is the collection [ID6]. In addition, unrelated individuals for cohort 1 are [ID2, ID3], whereas unrelated individuals for cohort 2 are [ID4, ID5].

Let $z_{1}^{u},z_{2}^{u}$ denote *z*-scores had GWAS been run using only unrelated individuals. Assume further that


$$z_{1}=\;\sqrt{1-\alpha_{1}}z_{1}^{u}+\sqrt{\alpha_{1}}z^{r}$$

$$z_{2}=\;\sqrt{1-\alpha_{2}}z_{2}^{u}+\sqrt{\alpha_{2}}z^{r}$$

where $\alpha_{i}=\hat{n_{12}}/n_{i}$ for $n_{i}=$ sample size for cohort *i* for i=1,2, and $z_{1}^{u},z_{2}^{u},z^{r}∼N(0,1)$. Notice that this assumption generalizes (sample-size based) standard meta-analysis method because we get $Cov(z_{1},z_{2})=0$ in the case of both cohorts consists of only unrelated individuals. Then

$Corr(z_{1},z_{2})=E(Z_{1}Z_{2})=\sqrt{\alpha_{1}\alpha_{2}}Var(z^{r})=\sqrt{\alpha_{1}\alpha_{2}}.$ Or equivalently, $Corr(z_{1},z_{2})=\hat{n_{12}}/(\sqrt{n_{1}n_{2}})$.

Suppose now that $\beta_{i},SE_{i}$ for i=1,2 are obtained for a nonnull SNP in the genotype data so that $z_{1},z_{2}∼N(c,1)$ for some c>0. Then, $z_{1}-c,\;z_{2}-c∼N(0,1)$. Following the same argument above, we get


$$z_{1}-c=\;\sqrt{1-\alpha_{1}}(z_{1}^{u}-c)+\sqrt{\alpha_{1}}(z^{r}-c)$$

$$z_{2}-c=\;\sqrt{1-\alpha_{2}}(z_{2}^{u}-c)+\sqrt{\alpha_{2}}(z^{r}-c)$$

where $\alpha_{i}=\hat{n_{12}}/n_{i}$ for $n_{i}=$sample size for cohort *i* for i=1,2, and $z_{1}^{u}-c,z_{2}^{u}-c,z^{r}-c∼N(0,1)$. Then $Corr(z_{1},z_{2})=Corr(z_{1}-c,z_{2}-c)=\sqrt{\alpha_{1}\alpha_{2}}.$ Or equivalently, $Corr(z_{1},z_{2})=\hat{n_{12}}/\sqrt{n_{1}n_{2}}$.

Hence, we can compute $\hat{n_{12}}$ by $\hat{n_{12}}=Corr(\beta_{1},\beta_{2})*\sqrt{n_{1}n_{2}}\;$ in either case. Combining this result with Equation 8 in Lin and Sullivan (2009), we get


(1) $$Corr(\beta_{1},\beta_{2})=Corr(z_{1},z_{2})$$

Finally, this factor becomes too conservative whenever $\beta_{1}*\beta_{2}<0$ (i.e. effect sizes are in different directions between cohorts). To address this issue, we add a calibration factor that we validated through extensive simulations to this equation such that


(2) $$Corr(\beta_{1},\beta_{2})=\gamma_{12}Corr(z_{1},z_{2})$$

where $\gamma_{12}=\{\begin{array}{cc} -1 & \mathrm{if}\;\beta_{1}*\beta_{2}<0\\ 1 & \mathrm{else} \end{array}$. Without this calibration factor we have not been able to get the desired accuracy from our estimations.

### Binary traits

Our proposed approach implements standard GWAS for each sub-cohort. Although the outlined method is developed for continuous phenotypes, we have tested it on binary traits and validated its accuracy and efficiency.

### Power-inflation feature

A fundamental step of our method is to estimate covariance terms in the associated variance estimations. Whenever those terms are positive, we have larger confidence intervals, which causes a drop in statistical power, albeit at a gain in FPR. As such, we decoupled the discovery and predictivity parts in our method. After estimating effect sizes correctly, our method inflates the associated test scores based on prior information about the heritability of the trait. This is an optional parameter for users who want to have similar statistical power and type I error rate as with standard GWAS approaches. Without this feature, our method still offers a better type I error rate, albeit at the cost of statistical power. We have developed this feature through simulation studies and validated it on both simulated and real data examples.

The intuition behind our argument is based on the fact that Median(TestScore)=1 after the genomic control step for standard GWAS. As such, we inflate test score according to a prior heritability estimation of the trait, because higher heritability indicates SNPs explain more of the phenotypic variation. We inflate the test score as a linear function of heritability. Precisely, the inflation of the test score is based on using a pre-determined heritability estimation, which can be obtained from previous studies such as Gene-Atlas (Canela-Xandri *et al.* 2018) and is followed by the following 2 steps:

*Step 1 (Genomic Control):*

$T\to \frac{T}{\lambda }$ where *λ* is the genomic control variable.

Step 2 (Inflation):

$T\to T*Max(1,\;0.95+h^{2})$ if trait is continuous,

$T\to T*Max(1,\;0.85+h^{2})$ if trait is binary.

### Heritability estimations

The power-inflation feature of CMA requires pre-predicted heritability estimations to enhance the accuracy of test statistics. These estimations can be obtained through online resources such as (Canela-Xandri *et al.* 2018) or derived using various methods. Examples include restricted maximum likelihood (REML)-based methods like GREML (also known as GCTA-REML) (Yang *et al*. 2010), GREML-LDMS (Yang *et al*. 2015), BOLT-REML (Loh *et al*. 2015), as well as Haseman–Elston regression-based approaches such as RHE-mc (randomized multicomponent version of the classical Haseman–Elston regression) (Pazokitoroudi *et al*. 2020) which is an extension of RHE-reg (randomized HE-regression) (Wu and Sankararaman 2018). Among these methods, RHE-mc stands out as the fastest, capable of delivering heritability estimates rapidly. For instance, RHE-mc can estimate approximately one hundred variance components—essential steps in heritability estimations—on datasets comprising a million individuals genotyped at one million SNPs in just a few hours (Pazokitoroudi *et al*. 2020).

### Choice of split index

For small sample studies, we recommend using exact methods. For larger studies, the choice of split index depends on user requirements such as memory efficiency, time efficiency, predictivity, or discovery.

In the context of the CMA method, which follows a meta-analysis approach, quality control steps like the “garbage out” principle are crucial (Borenstein *et al*. 2021). Studies with fewer than 15,000–20,000 individuals—whether or not they fall into the “garbage” category—often fail to identify a good level of discovery signals (Tam *et al*. 2019). Therefore, sub-cohort sizes that are too small are suboptimal for effective analysis. On the other hand, CMA benefits from between-study variance, which helps estimate confounding factors such as population structure and family-relatedness. To reliably capture between-study confounding effects, a sufficient number of sub-cohorts is necessary.

For our study based on the UKB cohort, we recommend sub-cohorts of 20,000–25,000 individuals. This range strikes a balance between statistical power, corresponding between-study variance, and computational efficiency. Notably, split indices like CMA-8 and CMA-16 tend to perform better than other alternative CMA methods in our simulation studies. We suggest that researchers adopt a similar balancing approach when deciding for ideal split index in their own studies.

### Dataset for predictivity and discovery analyses

We selected 409,204 individuals of white British ancestry and 488,928 variants in total from UKB, using mild filters on PLINK2 (Purcell *et al*. 2007; Chang *et al*. 2015). Precisely, individuals with genetic sex (UKB Data Field 22001) not consistent with their recorded sex (UKB Data Field 31) by UKB as well as individuals with missing genotype information of more than 10% were removed. Additionally, we applied a minor allele frequency filter of ≥1%, a minor allele count filter of 20, a Hardy–Weinberg equilibrium test not exceeding $P=1\;\times \;10^{-15}$, a genotyping rate above 99%, LD pruning using an $R^{2}$ threshold of 0.9 with a window size of 1,000 markers and a step size of 100 markers. Simulated phenotypes were generated for UKB individuals using genotypes to make simulations realistic. The covariates used in the corresponding GWAS models are age, age^2^, sex, and the top 20 principal components provided by the UKB.

### Data simulation for statistical performance on related individuals

We performed simulations to assess the empirical FPR and power of CMA under scenarios of unrelated and related individuals for both quantitative and binary traits. To mimic realistic scenarios, we used genotype array data from the UKB European samples and selected the white British samples, as in our predictivity analyses. We applied the same quality-control filters as in the predictivity analyses except for the LD pruning. We did not apply the pruning to evaluate false-positive rates on a higher number of markers. 505,977 SNPs passed the filters and were kept for the simulation.

From the UKB white British samples, we selected unrelated and related individuals, where we defined a pair of individuals as related if their estimated kinship coefficient, using the KING software (Manichaikul *et al*. 2010), was above 0.044, as provided by UKB and used in the REGENIE paper. We generated quantitative and binary traits using the GCTA software. We simulated up to 50 phenotypic replicates for each simulation setting. We selected 10,000, 30,000, or 50,000 SNPs to be causal for quantitative traits. For binary traits, the sample prevalence *K* was 0.1, 0.01, or 0.001, corresponding to a case-control ratio of 1:9, 1:99, or 1:999, and we fixed the number of causal SNPs to 10,000. For the scenario with only related individuals, we selected 1,000 causal variants and varied the case-control ratio between 1:9, 1:45, and 1:99 because of the smaller sample size.

The causal SNPs were randomly selected from odd chromosomes and with a minor allele count above 100. The effect sizes of the causal SNPs were sampled from a normal distribution with a mean of zero and the variance parameter was chosen to explain 20% of the trait variance ($h^{2}=0.2$). For binary traits, this variance was explained on the logistic scale. The environmental effects or residuals were sampled from a normal distribution with a mean of zero and the variance was set to correspond to a trait variance of one.

### Data simulation for 5 and 2 M individuals

To evaluate computational performance on data with population structure, we simulated a dataset of 5 million individuals from the HapMap3 (release 2 build 36) haplotype data using HAPGEN2 (Su *et al.* 2011). We applied the same QC filters as in the UKB dataset except for the LD pruning. For step 1 of REGENIE and R + CMA, we applied LD pruning using an $R^{2}$ threshold of 0.05 with a window size of 500 kb markers, which resulted in about 80,000 variants. For fastGWA-GLMM and F + CMA, we used a subset of 2 million individuals to limit the computation time for generating GRM. For step 2 in REGENIE and R + CMA, and GLM fitting in fastGWA-GLMM and F + CMA, we used 1 million variants resulting from LD pruning using an $R^{2}$ threshold of 0.9 with a window size of 1,000 markers and a step size of 100 markers.

### Computational efficiency of CMA

GWAS with large population cohorts is computationally demanding in terms of computational time and memory requirement. A common approach to reducing the memory requirement is to use a subset of the available markers or a subset of individuals. However, this may not capture highly significant markers and decrease statistical power. In contrast, CMA reduces the memory requirement by dividing a large cohort into smaller sub-cohorts and combining the analysis results of the sub-cohorts. In addition, by splitting a large cohort into smaller cohorts, CMA can achieve a higher computational efficiency from a more fine-grained workload partitioning.

Partitioning is necessary because processing the whole genotype matrix of millions of markers in a large population requires a large computer with large memory space. Recent methods (Jiang *et al*. 2021; Mbatchou *et al*. 2021) reduce the memory space by dividing a workload of the whole set of markers into workloads of smaller marker subsets. However, analyzing smaller subsets of markers will still need a large memory space if the sample size is large. In contrast, a workload with smaller subsets of markers and samples requires less memory space and can be run on smaller-scale and cheaper computers. Another advantage of dividing the cohort is that the workloads of different sub-cohorts can be computed in parallel to improve the utilization of processing units.

The advantages of the proposed method can be accomplished through 2 strategies: serial and parallel sub-cohort computations (Supplementary Fig. 20). The serial strategy computes different sub-cohorts sequentially to reduce the memory space required for analyzing the whole cohort. Alternatively, the parallel strategy computes different sub-cohorts in parallel to decrease the computation time. As the parallel computations need to load multiple sub-cohort data to memory simultaneously, the memory space used by the parallel strategy is higher than the sequential strategy and linearly increases with the number of sub-cohorts computed in parallel. However, this strategy can increase processing units and nodes utilization to reduce computation time. In cloud computing platforms, such as RAP, these strategies allow users to choose the compute instances according to their time and budget constraints.

Besides workload partitioning, the proposed method further saves computational resources by permitting incremental GWAS. Biobank datasets are usually published or generated incrementally as companies or projects generate genotype or sequence data as participants are added. Non-incremental GWAS protocol would analyze all samples at every release instead of incrementally adding evidence from new data, which leads to redundant computations. In contrast, CMA can incrementally generate association results as the data from added participants becomes available while appropriately accounting for potential relatedness.

### CMA implementation

We implemented CMA as 2 workflows in the Snakemake workflow system to adopt the serial and parallel strategies. R + CMA (Supplementary Fig. 21) uses steps 1 and 2 of REGENIE to fit a whole-genome regression model and performs single SNP association tests, respectively. F + CMA (Supplementary Fig. 22) uses fastGWA-GLMM to generate the sparse matrix (--make-bK-sparse) and to perform single SNP association tests with the general linear mixed model (GLMM).

In R + CMA, REGENIE's step 1 and step 2 routines are run independently for different sub-cohorts as jobs. On the other hand, F + CMA runs --make-bK-sparse and GLMM routines independently for different sub-cohort as jobs. These jobs can be executed in serial or parallel by selecting an option when launching the workflow. For both workflows, the CMA component combines the sub-cohort results to generate the final GWAS statistic results.

To divide the cohort into sub-cohorts, we implemented a cohort splitter component. We implement CMA and cohort splitter in Python and use the Numpy (Harris *et al*. 2020) and Scipy (Virtanen *et al*. 2020) libraries for optimized linear algebra computations. The steps 1 and 2 routines for association testing of each sub-cohort are invoked by running the REGENIE software, while the fastGWA-GLMM routines are invoked by running the GCTA software. To increase CPUs’ utilization without over-utilizing them, we schedule parallel computations by allocating different subsets of CPUs to different sub-cohort workloads (Supplementary Fig. 16b).

The computational complexity of the CMA implementations consists of 3 parts: splitting the cohort, computing association tests for each sub-cohort, and CMA computation. Cohort splitting has a time complexity of O(N), where *N* is the number of individuals. For CMA computation, the complexity stems from matrix multiplication and inverse operations. The time complexities of matrix multiplication and inverse computation are $O(m\;k^{2})$ and $O(k^{3})$, respectively, where *m* is the number of markers and *k* is the split index.

The time complexity of association testing is determined by the method used for the implementation, i.e. REGENIE and fastGWA-GLMM. However, CMA reduces the space complexity of the association testing from O(nm) to O((n/k)m) in serial computation, where n×m is the input size of the genotype matrix of the whole cohort. The parallel implementation of CMA has a space complexity of O((n/k)mp), where *p* is the number of sub-cohorts computed in parallel.

## Supplementary Material

iyaf019_Supplementary_Data

## Data Availability

The genotype and real phenotype data are accessible through formal application to the UK Biobank (http://www.ukbiobank.ac.uk). Simulated phenotypes are generated through standard simulation steps by GCTA (https://yanglab.westlake.edu.cn/software/gcta/#GWASSimulation). Our software (CMA) is available at https://git.ecdf.ed.ac.uk/cma/snake-cma. Supplemental material available at GENETICS online.
