# Supplementary figures and images for: Divide and conquer approach for genome-wide association studies
Source: Genetics. 2025 Mar 13;229(4):iyaf019. doi: 10.1093/genetics/iyaf019 (PMC12005250; doi:10.1093/genetics/iyaf019)

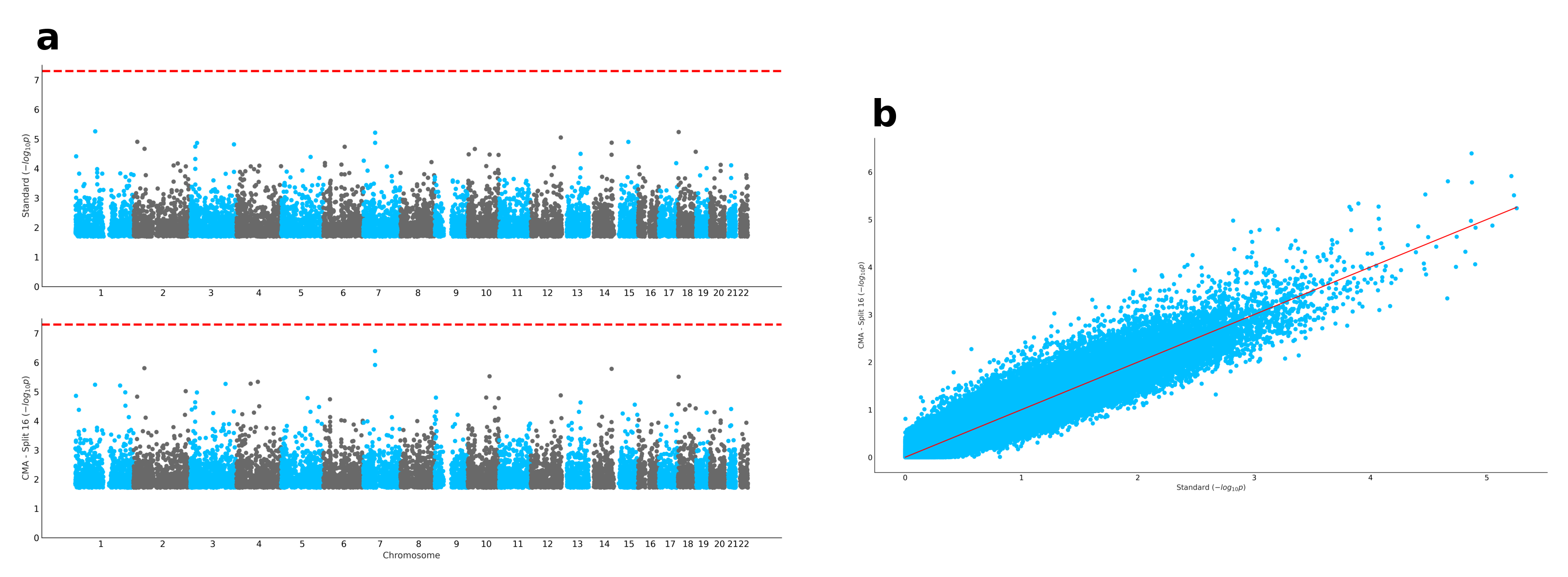

Supplement: iyaf019_Supplementary_Data [file iyaf019_supplementary_data.zip › Figure_S10_GENETICS-2024-307695.png]

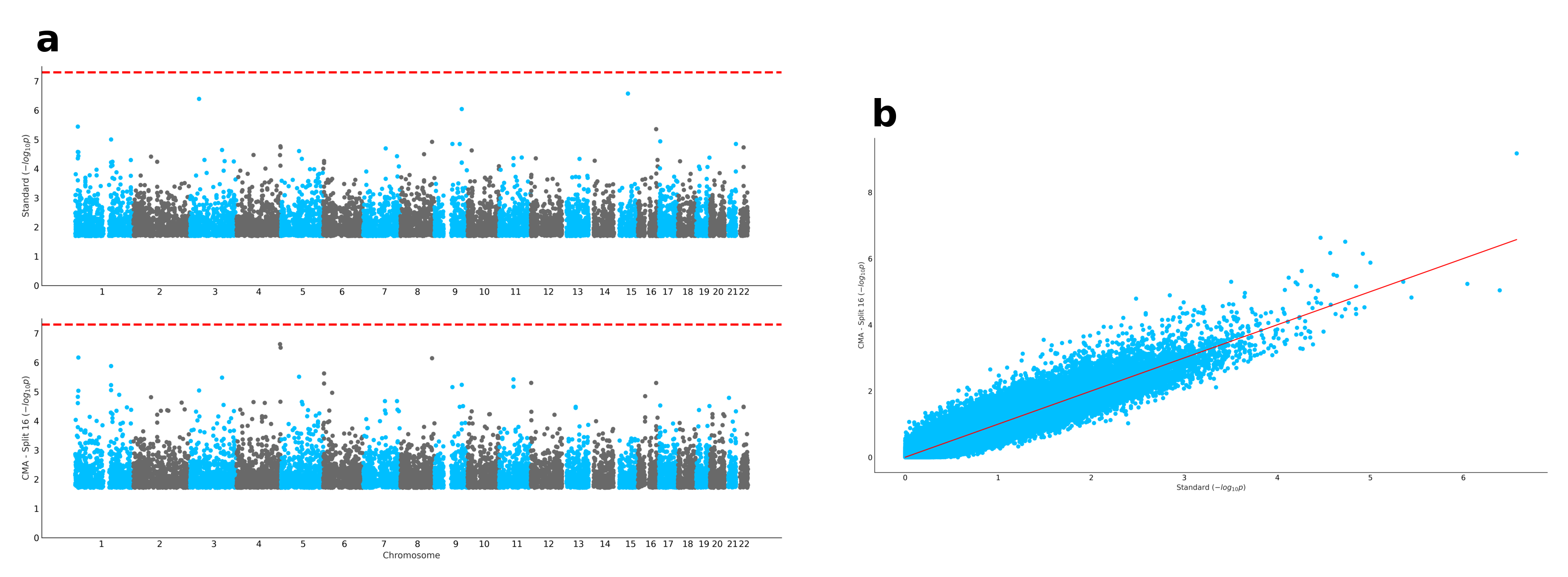

Supplement: iyaf019_Supplementary_Data [file iyaf019_supplementary_data.zip › Figure_S11_GENETICS-2024-307695.png]

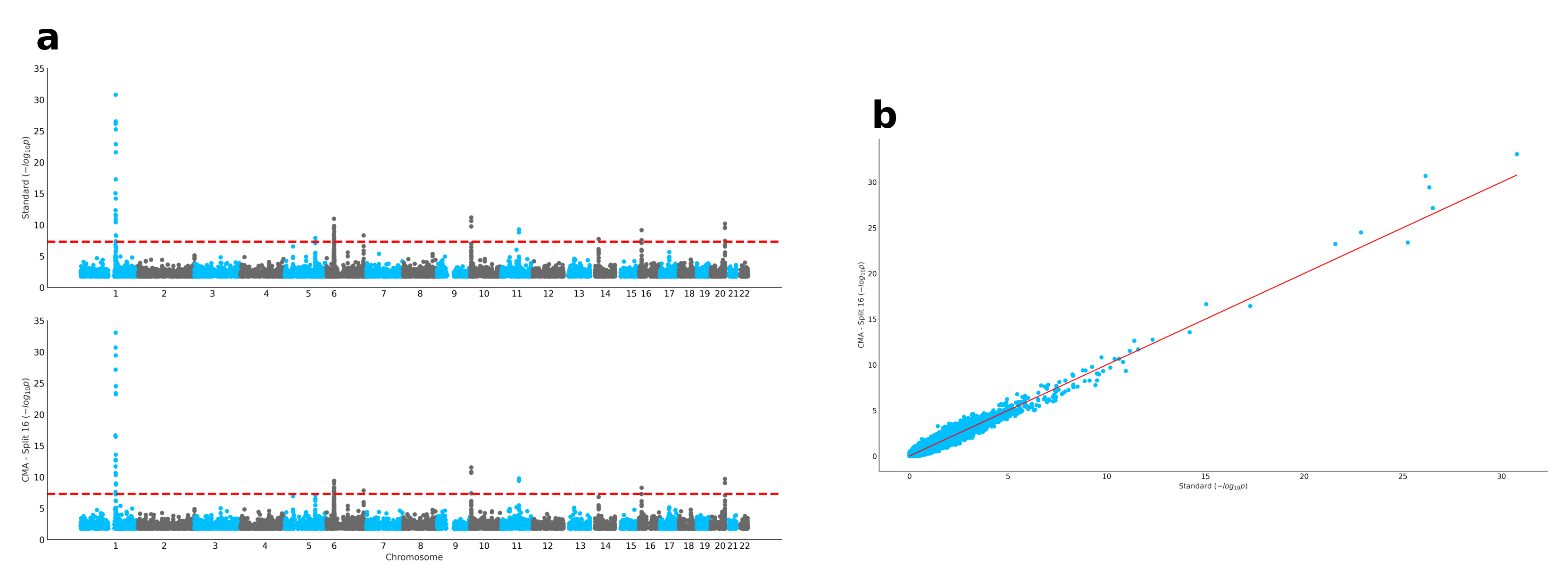

Supplement: iyaf019_Supplementary_Data [file iyaf019_supplementary_data.zip › Figure_S12_GENETICS-2024-307695.png]

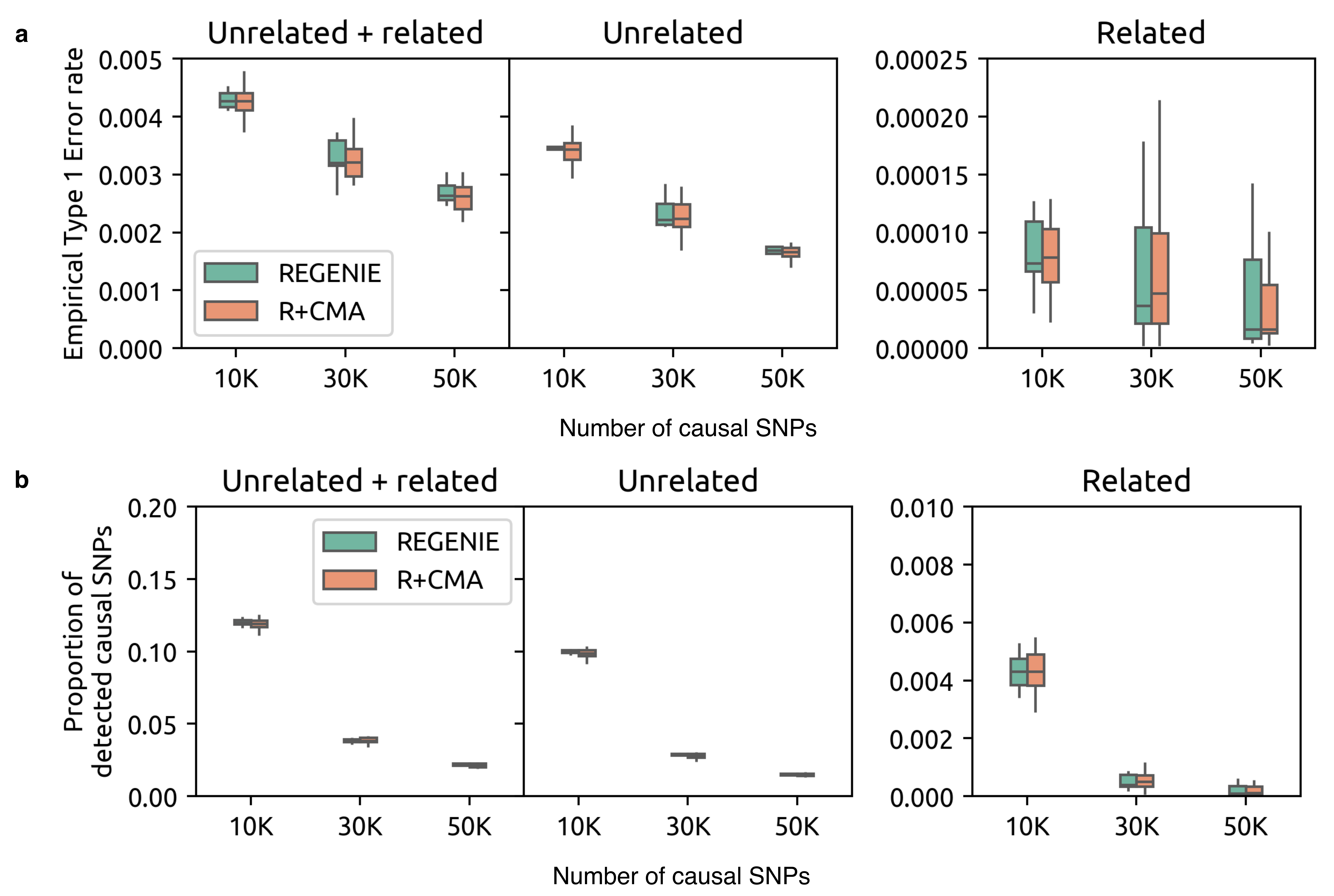

Supplement: iyaf019_Supplementary_Data [file iyaf019_supplementary_data.zip › Figure_S13_GENETICS-2024-307695.png]

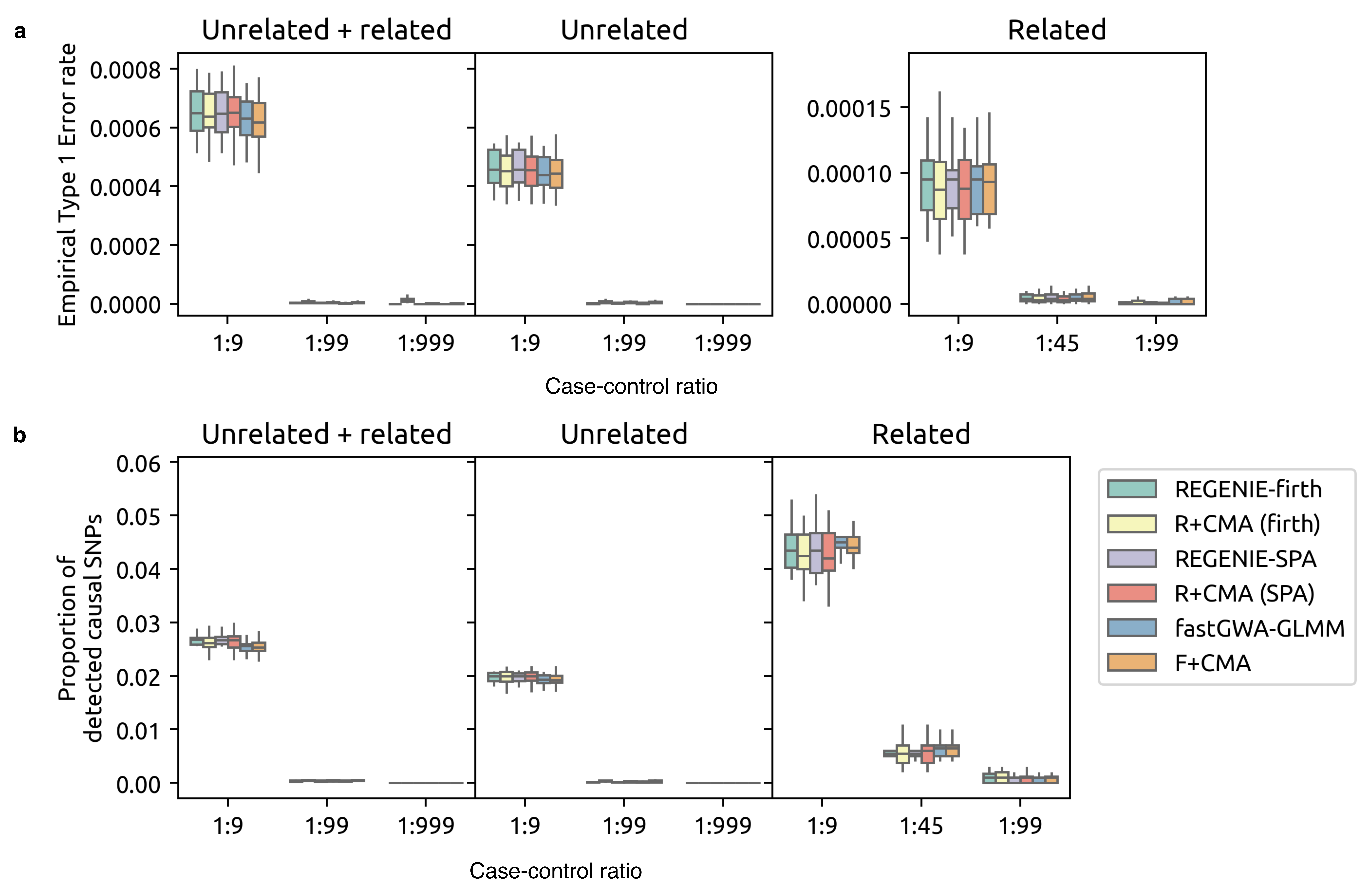

Supplement: iyaf019_Supplementary_Data [file iyaf019_supplementary_data.zip › Figure_S14_GENETICS-2024-307695.png]

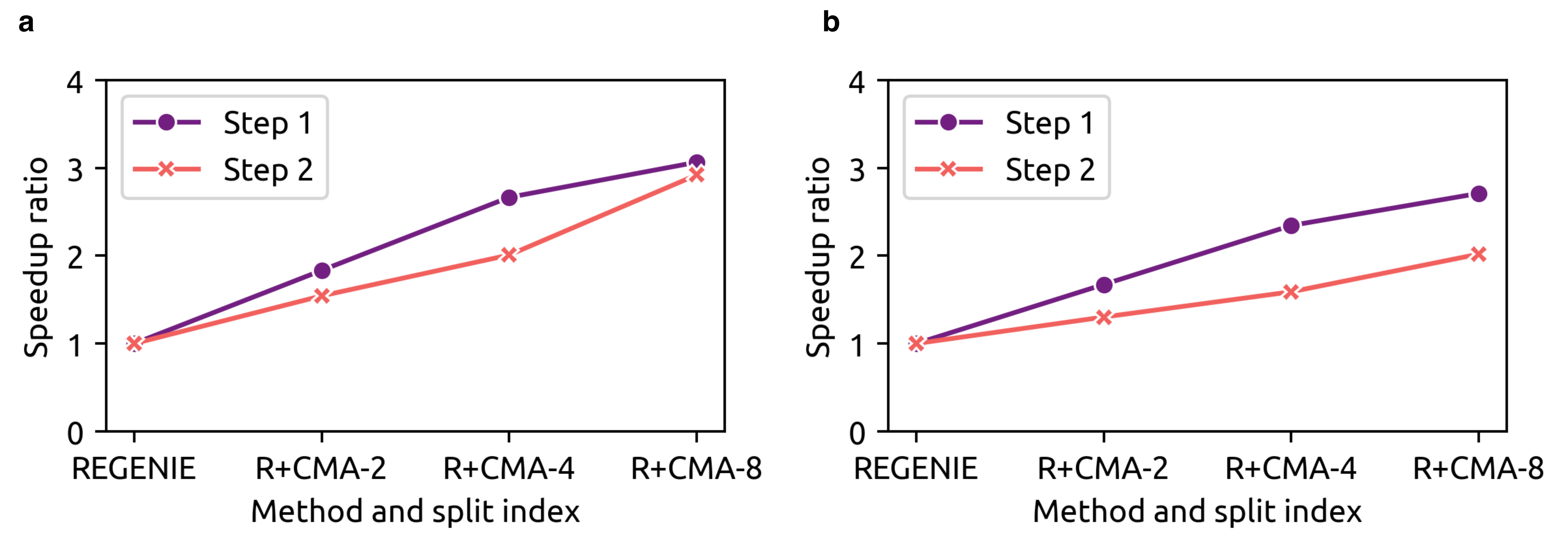

Supplement: iyaf019_Supplementary_Data [file iyaf019_supplementary_data.zip › Figure_S15_GENETICS-2024-307695.png]

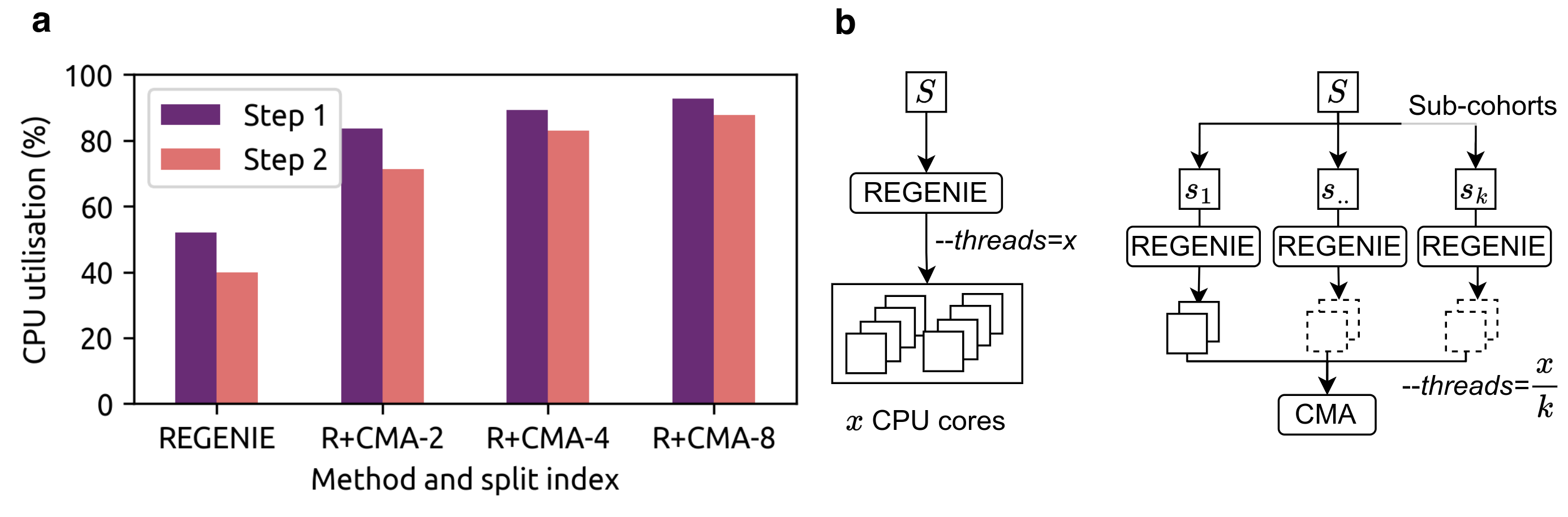

Supplement: iyaf019_Supplementary_Data [file iyaf019_supplementary_data.zip › Figure_S16_GENETICS-2024-307695.png]

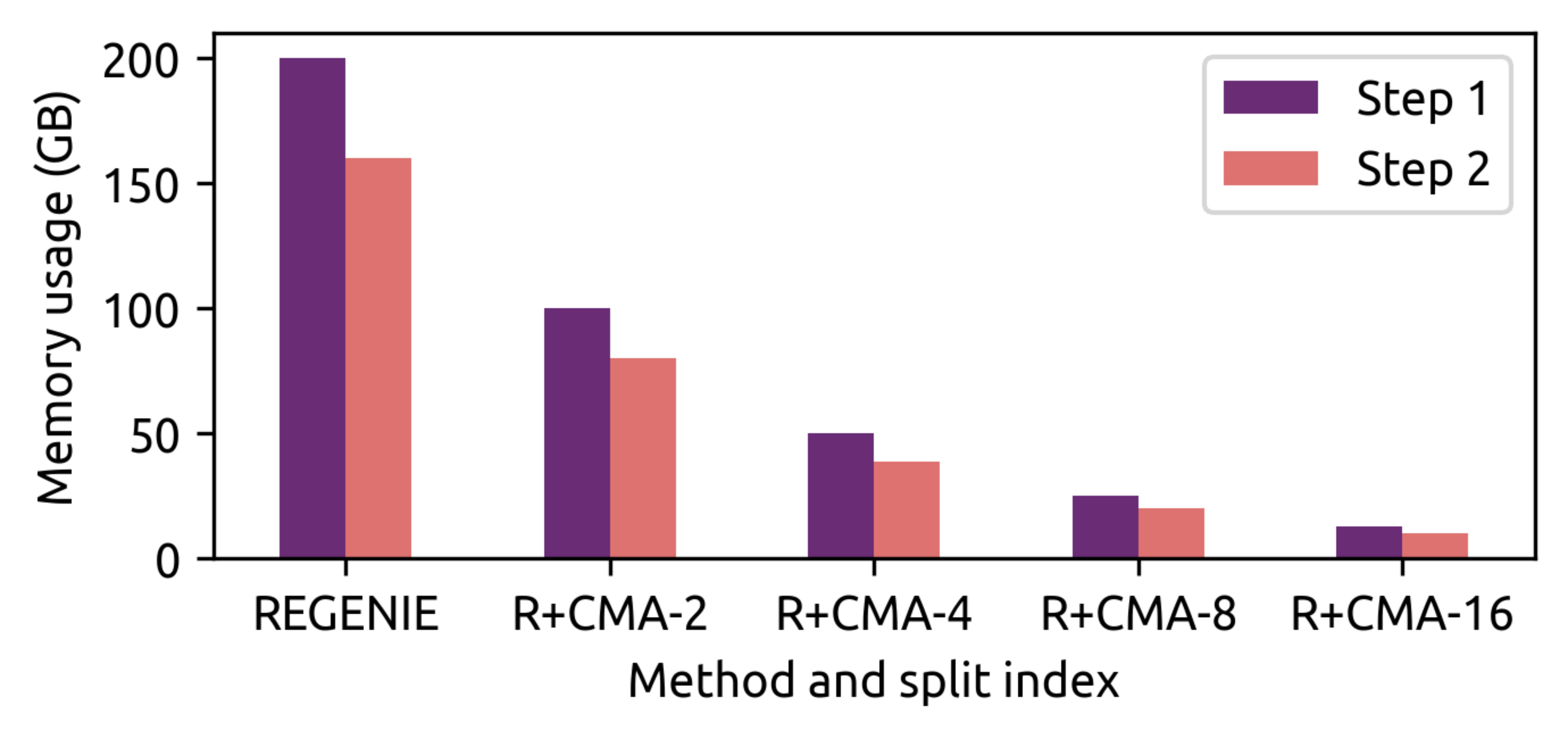

Supplement: iyaf019_Supplementary_Data [file iyaf019_supplementary_data.zip › Figure_S17_GENETICS-2024-307695.png]

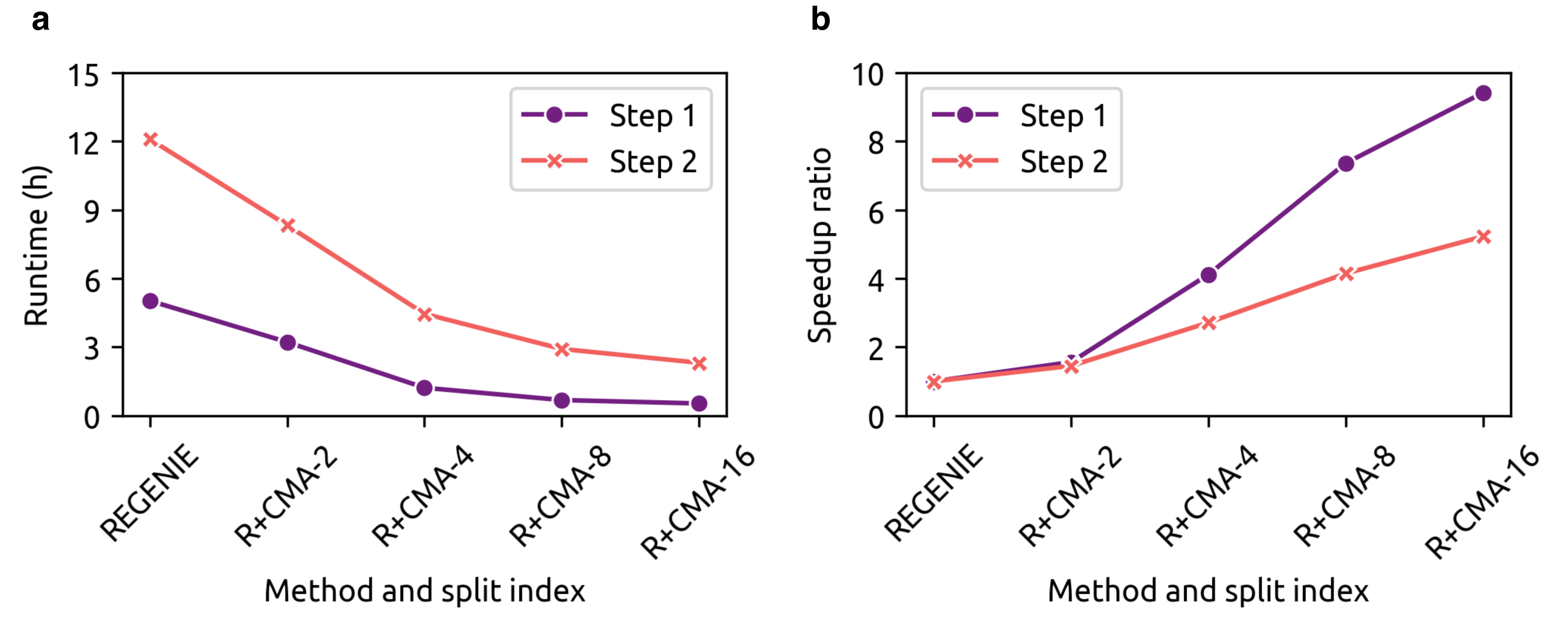

Supplement: iyaf019_Supplementary_Data [file iyaf019_supplementary_data.zip › Figure_S18_GENETICS-2024-307695.png]

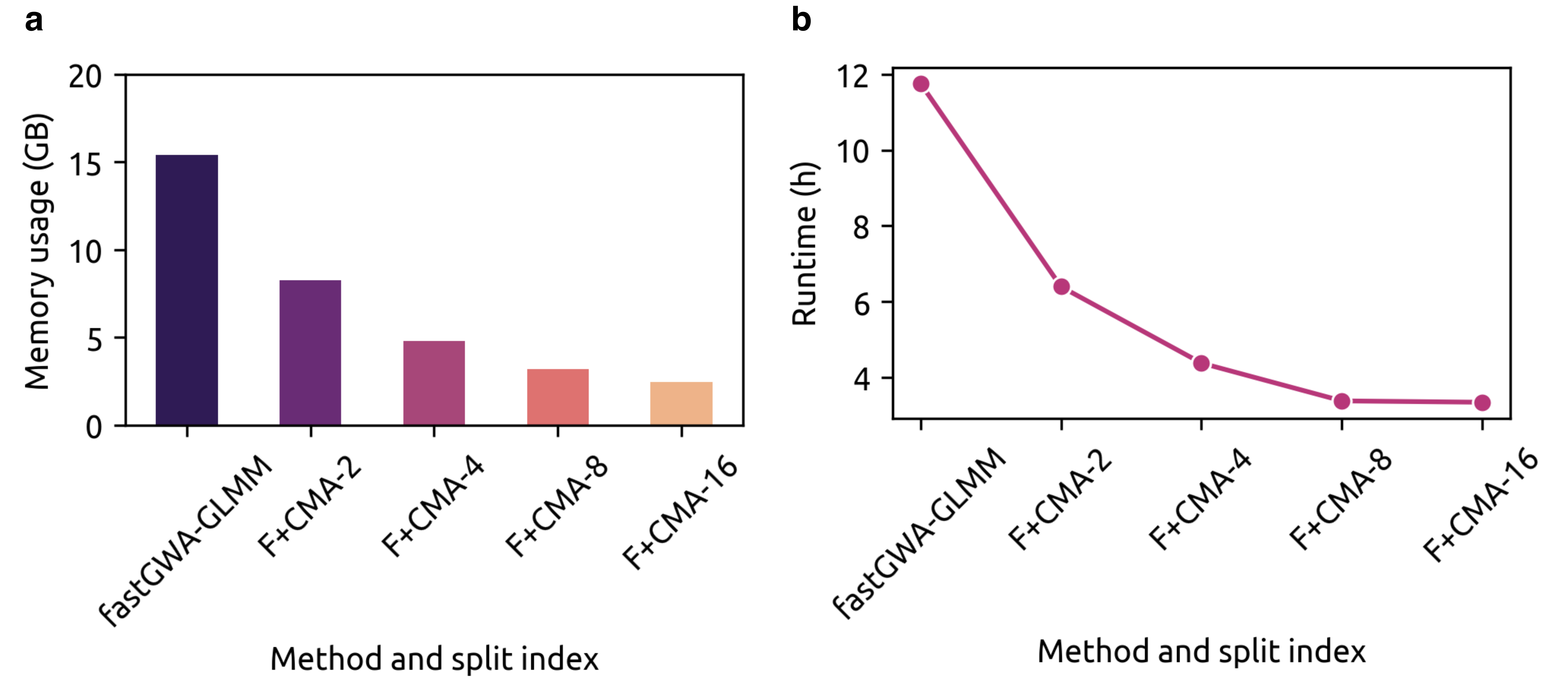

Supplement: iyaf019_Supplementary_Data [file iyaf019_supplementary_data.zip › Figure_S19_GENETICS-2024-307695.png]

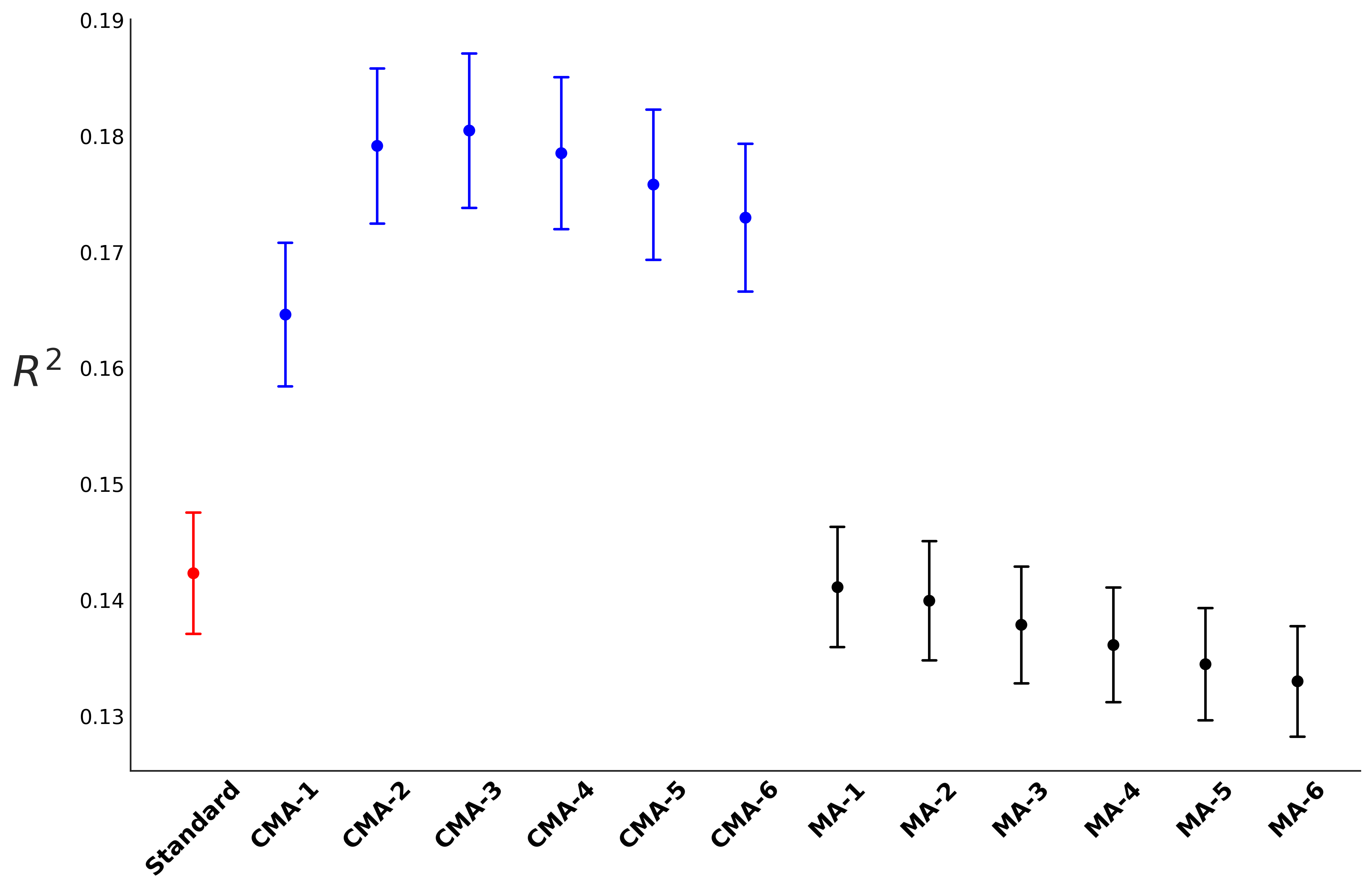

Supplement: iyaf019_Supplementary_Data [file iyaf019_supplementary_data.zip › Figure_S1_GENETICS-2024-307695.png]

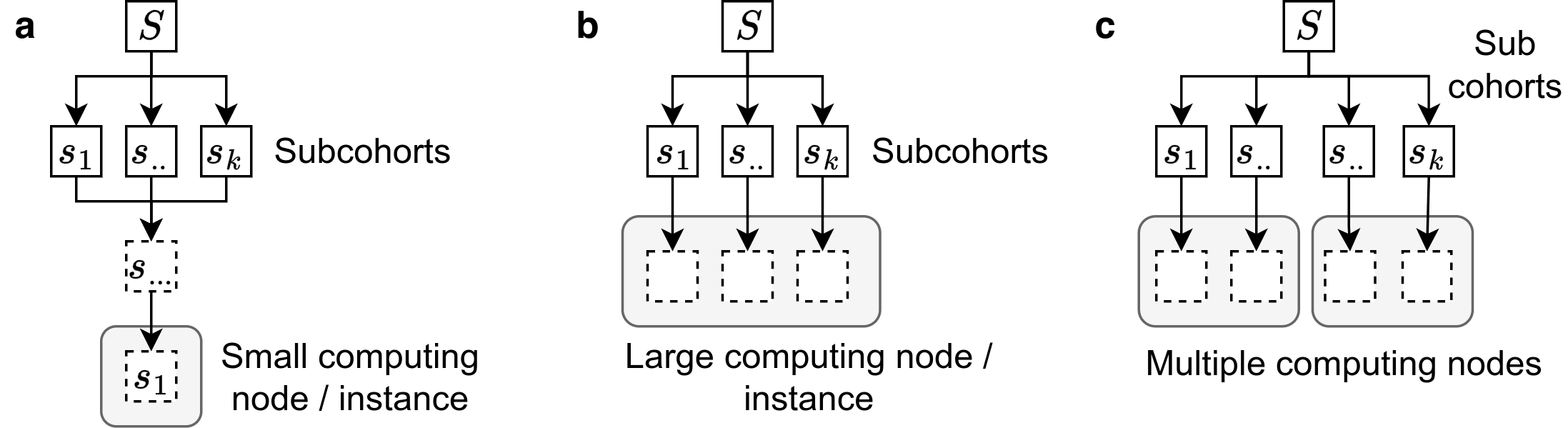

Supplement: iyaf019_Supplementary_Data [file iyaf019_supplementary_data.zip › Figure_S20_GENETICS-2024-307695.png]

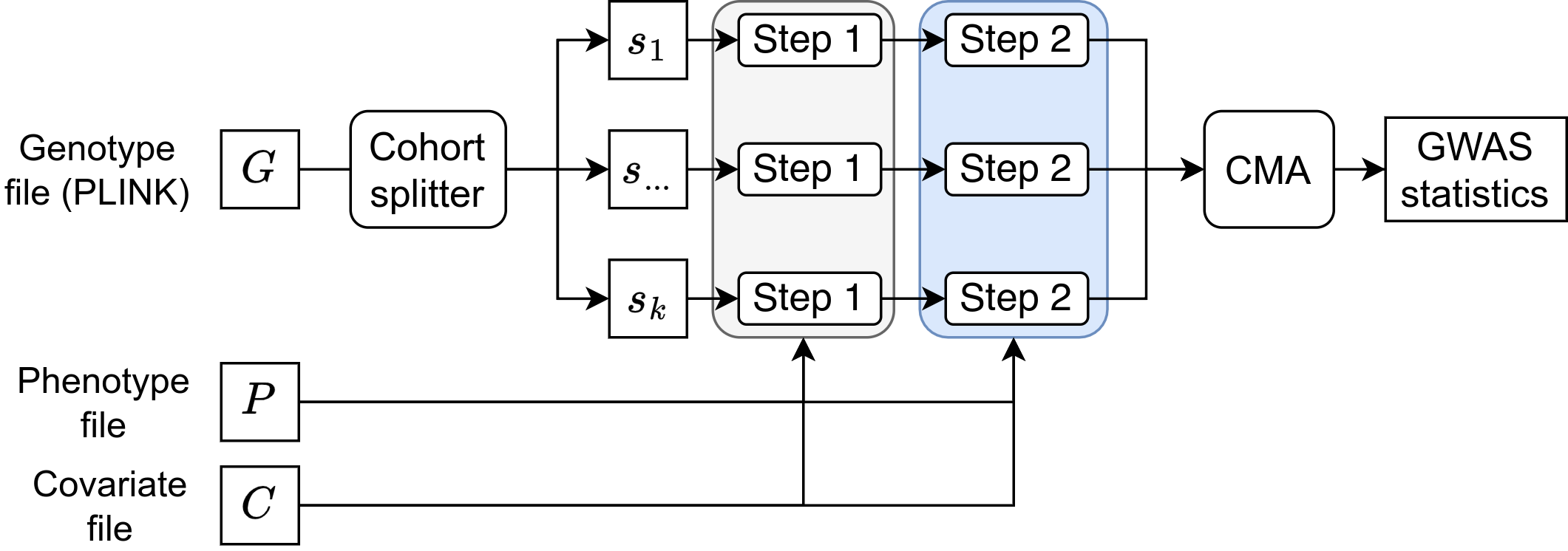

Supplement: iyaf019_Supplementary_Data [file iyaf019_supplementary_data.zip › Figure_S21_GENETICS-2024-307695.png]

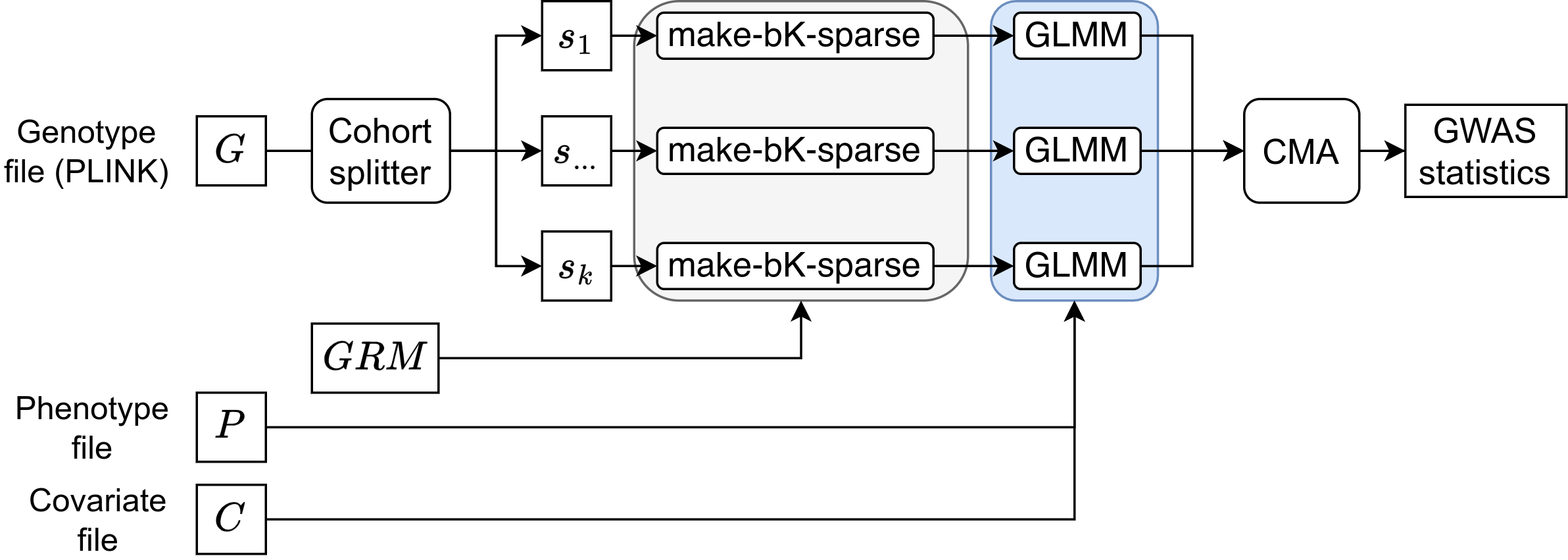

Supplement: iyaf019_Supplementary_Data [file iyaf019_supplementary_data.zip › Figure_S22_GENETICS-2024-307695.png]

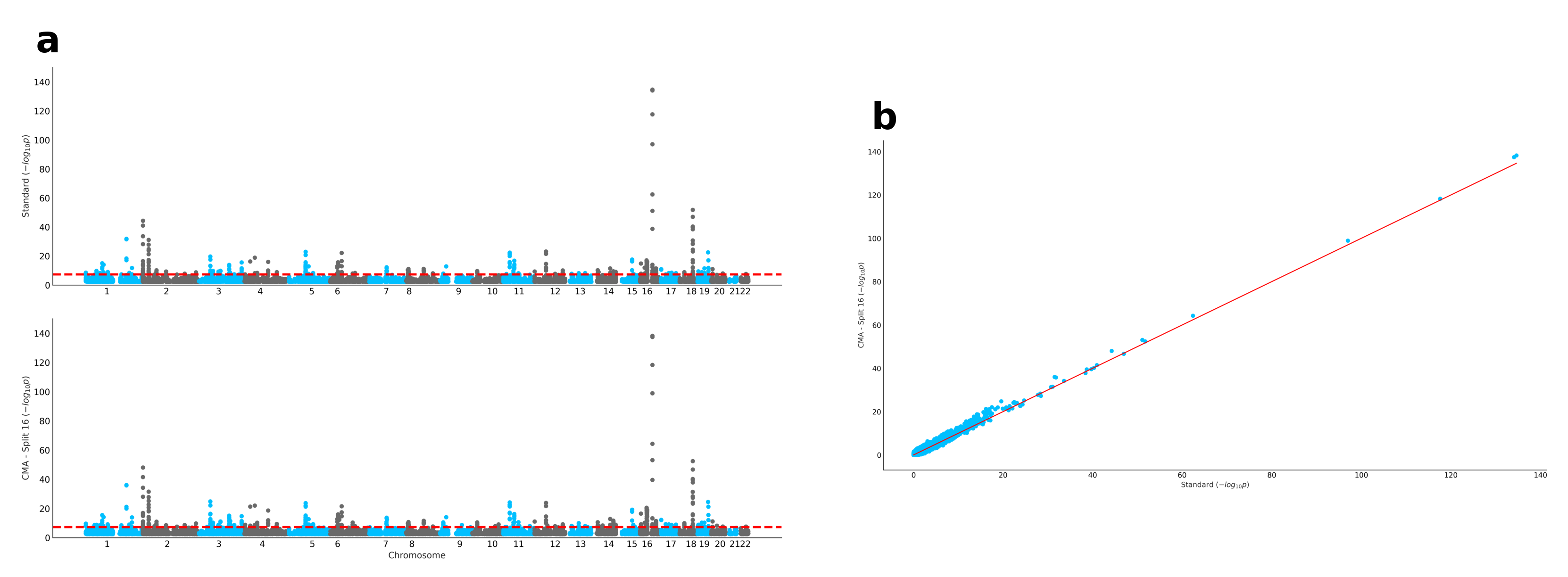

Supplement: iyaf019_Supplementary_Data [file iyaf019_supplementary_data.zip › Figure_S2_GENETICS-2024-307695.png]

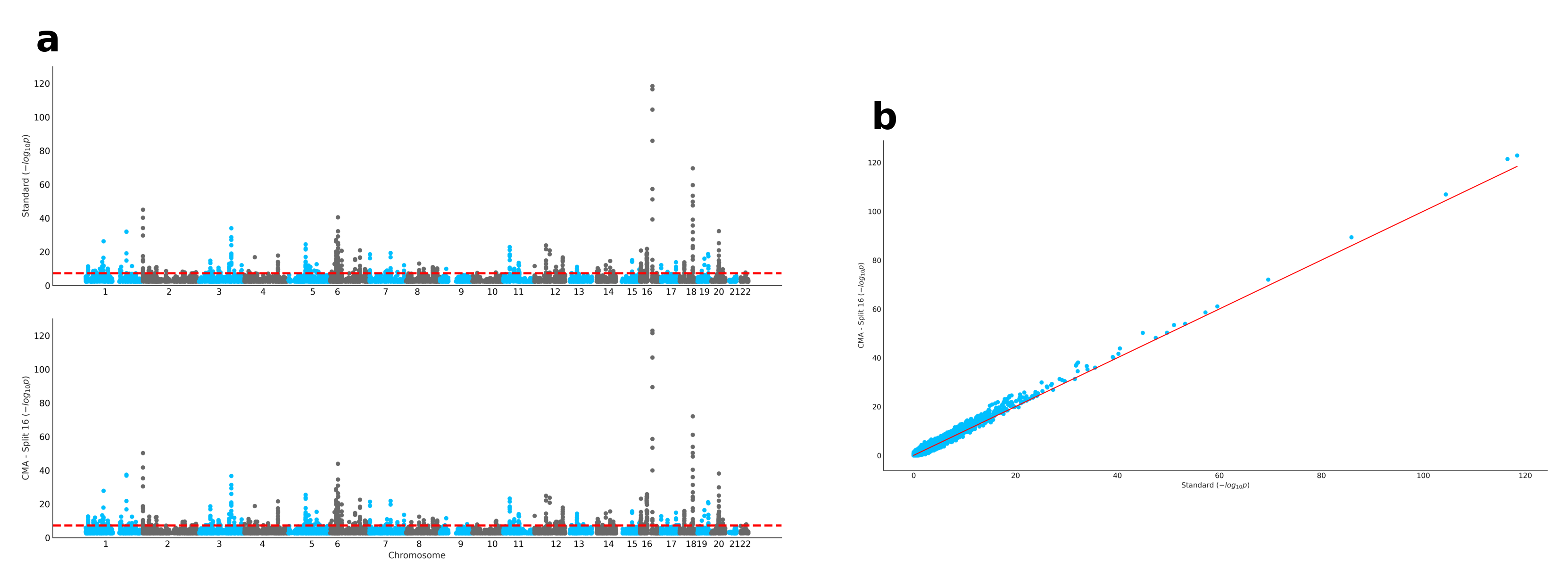

Supplement: iyaf019_Supplementary_Data [file iyaf019_supplementary_data.zip › Figure_S3_GENETICS-2024-307695.png]

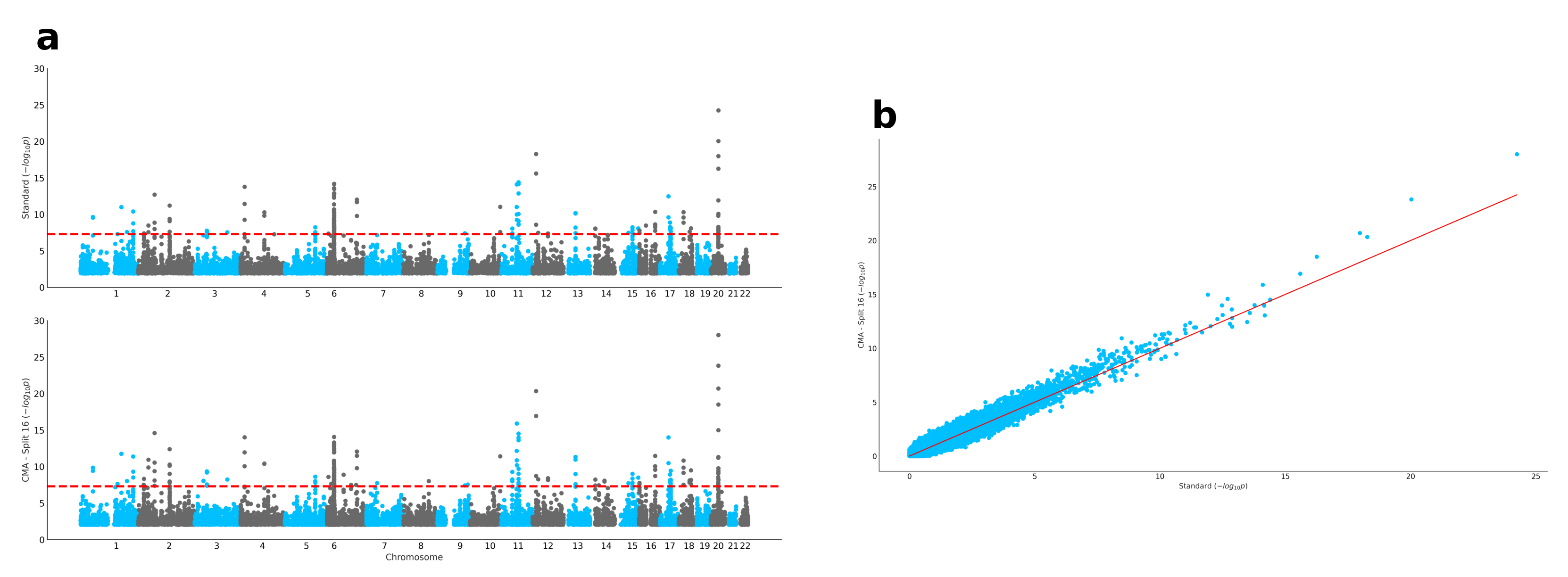

Supplement: iyaf019_Supplementary_Data [file iyaf019_supplementary_data.zip › Figure_S4_GENETICS-2024-307695.png]

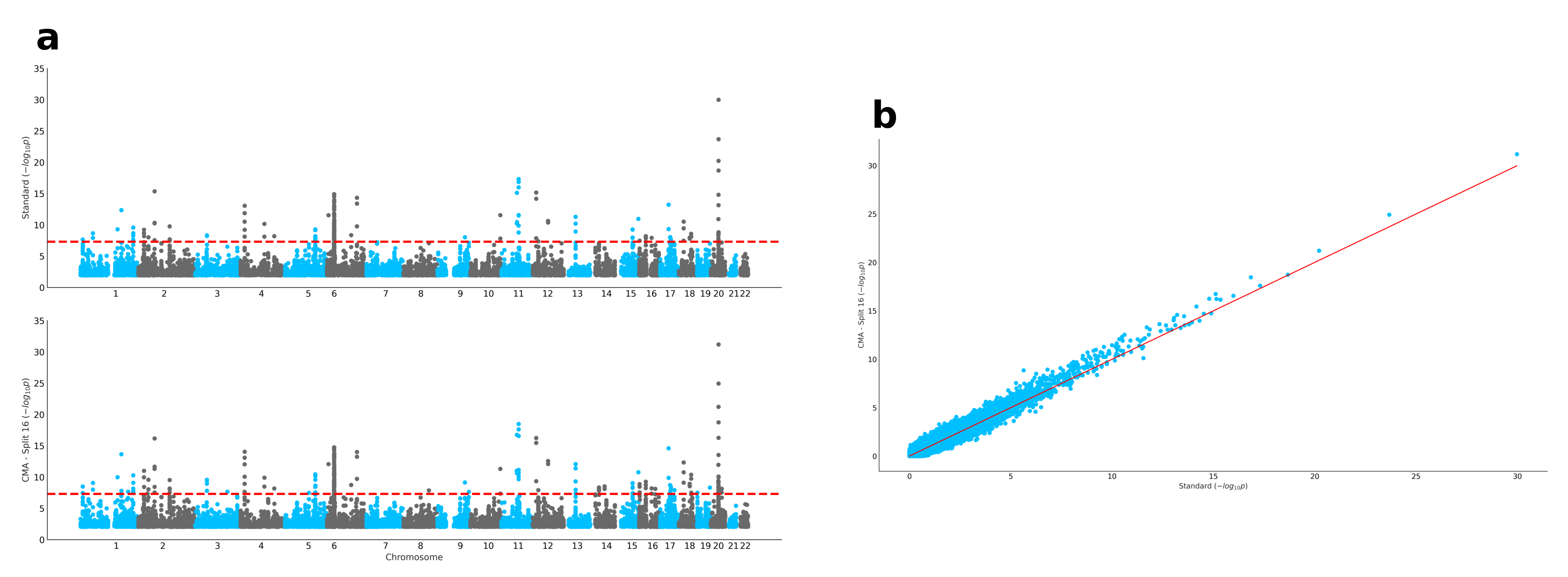

Supplement: iyaf019_Supplementary_Data [file iyaf019_supplementary_data.zip › Figure_S5_GENETICS-2024-307695.png]

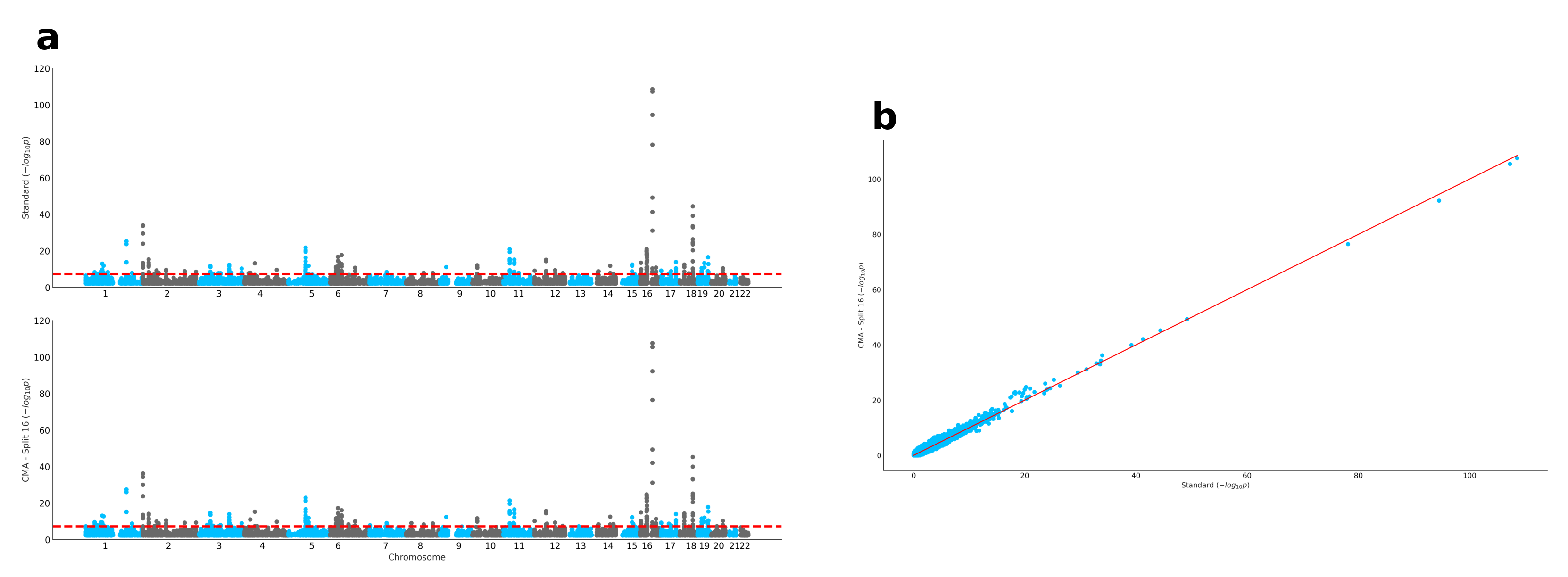

Supplement: iyaf019_Supplementary_Data [file iyaf019_supplementary_data.zip › Figure_S6_GENETICS-2024-307695.png]

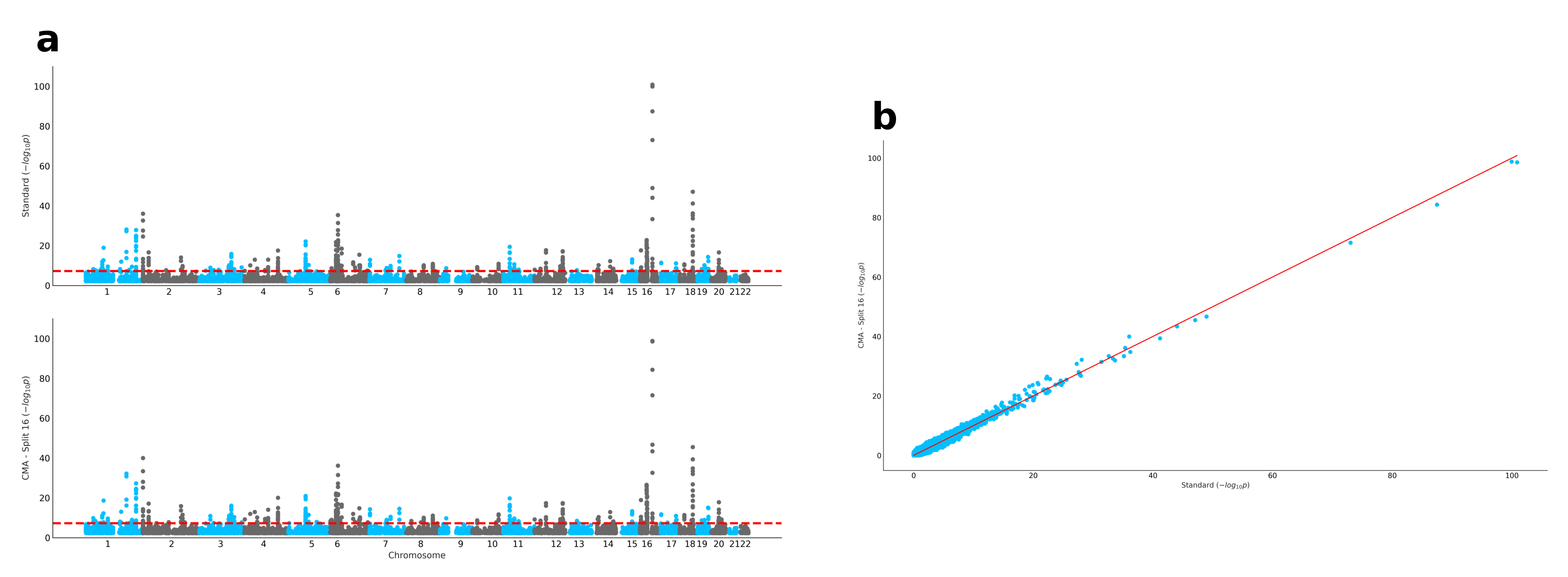

Supplement: iyaf019_Supplementary_Data [file iyaf019_supplementary_data.zip › Figure_S7_GENETICS-2024-307695.png]

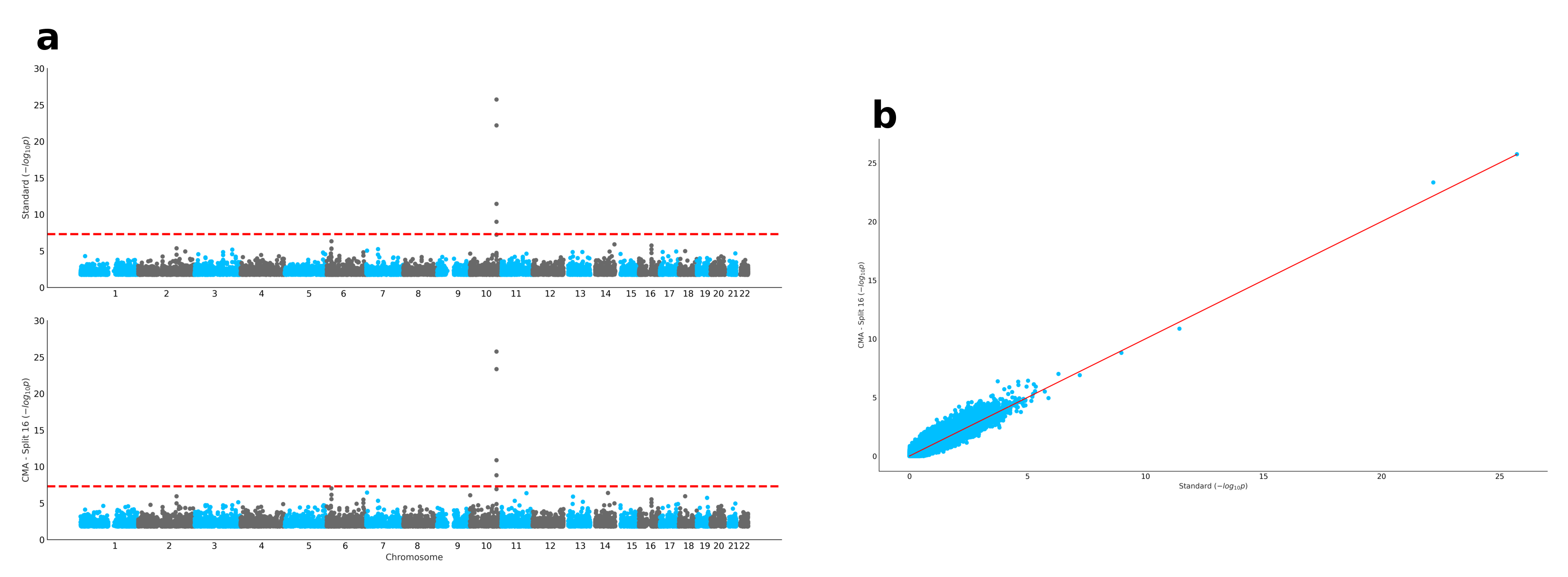

Supplement: iyaf019_Supplementary_Data [file iyaf019_supplementary_data.zip › Figure_S8_GENETICS-2024-307695.png]

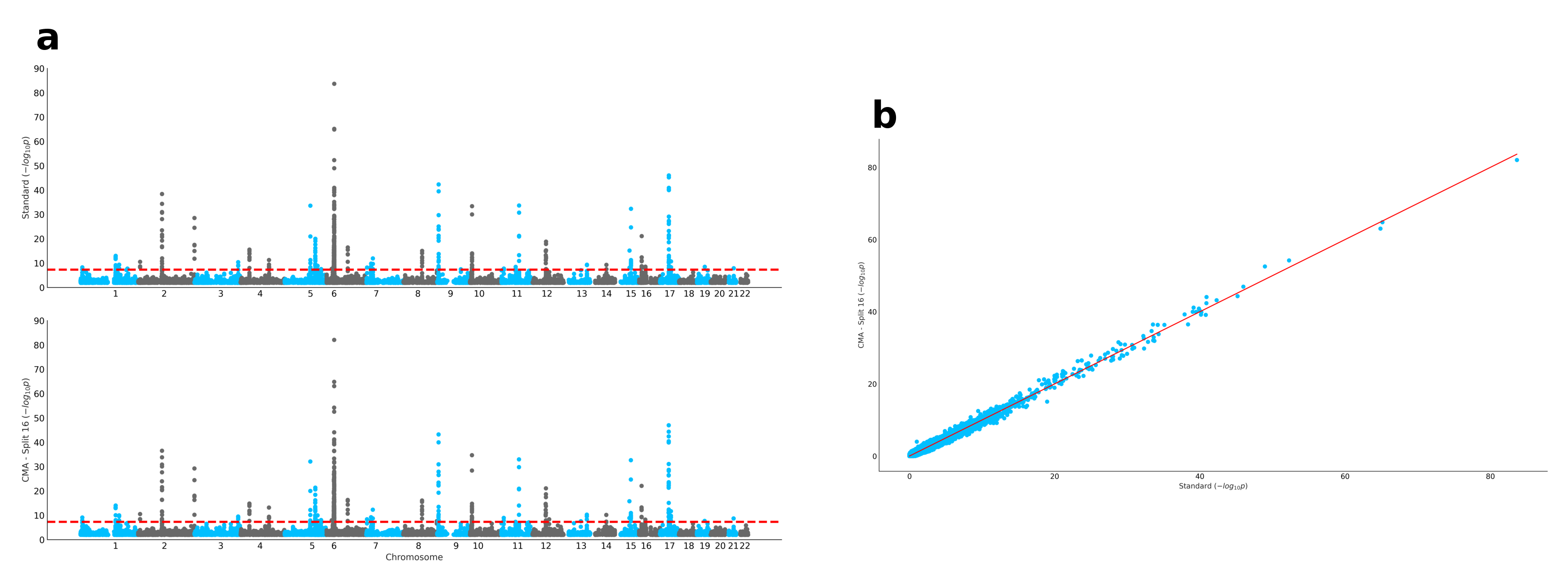

Supplement: iyaf019_Supplementary_Data [file iyaf019_supplementary_data.zip › Figure_S9_GENETICS-2024-307695.png]
